# National survey on the prevalence of single-gene aetiologies for genetic developmental and epileptic encephalopathies in Italy

**DOI:** 10.1136/jmg-2024-110328

**Published:** 2024-11-28

**Authors:** Davide Mei, Simona Balestrini, Elena Parrini, Antonio Gambardella, Grazia Annesi, Valentina De Giorgis, Simone Gana, Maria Teresa Bassi, Claudio Zucca, Maurizio Elia, Luigi Vetri, Barbara Castellotti, Francesca Ragona, Mario Mastrangelo, Francesco Pisani, Giuseppe d'Orsi, Massimo Carella, Dario Pruna, Sabrina Giglio, Carla Marini, Elisabetta Cesaroni, Antonella Riva, Marcello Scala, Laura Licchetta, Raffaella Minardi, Ilaria Contaldo, Maria Luigia Gambardella, Alberto Cossu, Jacopo Proietti, Gaetano Cantalupo, LICE Collaborative Group, Elena Cellini, Marina Trivisano, Angela De Dominicis, Nicola Specchio, Laura Tassi, Renzo Guerrini

**Affiliations:** 1 Neuroscience and Human Genetics Department, Meyer Children's Hospital IRCSS, Florence, Italy; 2 University of Florence, Florence, Italy; 3 Dipartimento di Scienze Mediche e Chirurgiche, Università degli Studi Magna Graecia, Catanzaro, Italy; 4 Institute for Biomedical Research and Innovation, National Research Council, Cosenza, Italy; 5 Brain and Behavioral Sciences Department, University of Pavia, Pavia, Italy; 6 Childhood and Adolescence Epilepsy Center, Department of Child Neurology and Psychiatry, IRCCS Mondino Foundation, ERN EpiCARE Full Member, Pavia, Italy; 7 Neurogenetics Research Center, IRCCS Mondino Foundation, ERN EpiCARE Full Member, Pavia, Italy; 8 Laboratory of Genetics, Scientific Institute IRCCS Eugenio Medea, Bosisio Parini, Bosisio Parini, Italy; 9 Clinical Neurophysiology Unit, Scientific Institute IRCCS E. Medea, Bosisio Parini, Italy; 10 Oasi Research Institute - IRCCS, Troina, Italy; 11 Unit of Medical Genetics and Neurogenetics, Fondazione IRCCS Istituto Neurologico Carlo Besta, Milano, Italy; 12 Department of Pediatric Neuroscience, Fondazione IRCCS Istituto Neurologico Carlo Besta, Milano, Italy; 13 Department of Women/Child Health and Urological Sciences, Sapienza University of Rome, Rome, Italy; 14 Unit of Child Neurology and Psychiatry-Department of Neurosciences/Mental Health, Azienda Ospedaliero-Universitaria Policlinico Umberto I, Rome, Italy; 15 Department of Human Neuroscience, Sapienza University of Rome, Rome, Italy; 16 Fondazione IRCCS Casa Sollievo della Sofferenza, San Giovanni Rotondo, Foggia, Italy; 17 Child Neurology and Epileptology, S. Michele Hospital, ASL Cagliari, Cagliari, Italy; 18 Medical Genetics, R. Binaghi Hospital, ASL Cagliari, Cagliari, Italy; 19 Child neurology and psychiatric unit, Pediatric Hospital G. Salesi; AOU delle Marche, Ancona, Italy; 20 IRCCS Istituto Giannina Gaslini, Full Member of European Reference Network EpiCARE, Genova, Italy; 21 Department of Neurosciences, Rehabilitation, Ophthalmology, Genetics, Maternal and Child Health, University of Genova, Genova, Italy; 22 IRCCS Istituto delle Scienze Neurologiche di Bologna, IRCCS Istituto Delle Scienze Neurologiche di Bologna, Full member of the ERN EpiCARE, Bologna, Italy; 23 Child Neurology and Psychiatric Unit, Fondazione Policlinico Universitario Agostino Gemelli, IRCCS, Rome, Italy; 24 UOC Neuropsichiatria Infantile, Ospedale della Donna e del Bambino c/o Ospedale Civile Maggiore, AOUI Verona, Full member of ERN EpiCARE, Verona, Italy; 25 Department of Engineering for Innovation Medicine, University of Verona, Verona, Italy; 26 Neurology, Epilepsy and Movement Disorders Unit, Bambino Gesù Children's Hospital, IRCCS, Full Member of ERN EpiCARE, Rome, Italy; 27 Claudio Munari Epilepsy Surgery Center, ASST Grande Ospedale Metropolitano Niguarda, Milan, Italy

**Keywords:** Epilepsy, Genomics

## Abstract

**Background:**

We aimed to estimate real-world evidence of the prevalence rate of genetic developmental and epileptic encephalopathies (DEEs) in the Italian population over a 11-year period.

**Methods:**

Fifteen paediatric and adult tertiary Italian epilepsy centres participated in a survey related to 98 genes included in the molecular diagnostic workflows of most centres. We included patients with a clinical diagnosis of DEE, caused by a pathogenic or likely pathogenic variant in one of the selected genes, with a molecular diagnosis established between 2012 and 2022. These data were used as a proxy to estimate the prevalence rate of DEEs.

**Results:**

We included 1568 unique patients and found a mean incidence proportion of 2.6 patients for 100.000 inhabitants (SD=1.13) with consistent values across most Italian regions. The number of molecular diagnoses showed a continuing positive trend, resulting in more than a 10-fold increase between 2012 and 2022. The mean age at molecular diagnosis was 11.2 years (range 0–75). Pathogenic or likely pathogenic variants in genes with an autosomal dominant inheritance pattern occurred in 77% (n=1207) patients; 17% (n=271) in X-linked genes and 6% (n=90) in genes with autosomal recessive inheritance. The most frequently reported genes in the survey were *SCN1A* (16%), followed by *KCNQ2* (5.6%) and *SCN2A* (5%).

**Conclusion:**

Our study provides a large dataset of patients with monogenic DEE, from a European country. This is essential for informing decision-makers in drug development on the appropriateness of initiatives aimed at developing precision medicine therapies and is instrumental in implementing disease-specific registries and natural history studies.

**WHAT IS ALREADY KNOWN ON THIS TOPIC**

- Epidemiological data gathered from the real-world evidence are fundamental to assess the potential for genetically driven precision medicine and to prioritise investments in innovative therapies in developmental and epileptic encephalopathies (DEEs).

**WHAT THIS STUDY ADDS**

- This study aimed to determine the prevalence of genetic DEEs in Italy over an 11-year period, using molecular diagnoses as a proxy. We found that DEEs affected 2.6 per 100 000 inhabitants, with a consistent prevalence across different regions of Italy. The data revealed a significant increase in diagnoses over the decade, with an average age at diagnosis of 11.2 years. Most cases were linked to autosomal dominant genes (77%), followed by X-linked (17%) and autosomal recessive genes (6%). The genes *SCN1A*, *KCNQ2* and *SCN2A* emerged as the most frequently involved in DEEs.

**HOW THIS STUDY MIGHT AFFECT RESEARCH, PRACTICE OR POLICY**

- This comprehensive dataset from Italy, though not exhaustive, is crucial for guiding the development of targeted therapies, advancing precision medicine, and establishing disease-specific registries and natural history studies.

## Introduction

 The term ‘developmental and epileptic encephalopathies’ (DEEs) is used to designate disorders with early-onset severe epilepsy and EEG abnormalities on a background of developmental impairment that tends to worsen as a consequence of epilepsy.^1^ The associated clinical picture is complex and severely disabling, resulting from a combination of developmental abnormalities and severe epilepsy/EEG discharges.

Epidemiological data gathered from the real-world-evidence are fundamental to assess the potential for genetically driven precision medicine and to prioritise investments in innovative therapies in DEEs, as also advocated by the European Medicine Agency and national regulatory bodies (https://www.ema.europa.eu/en/reflection-paper-use-real-world-data-non-interventional-studies-generate-real-world-evidence-scientific-guideline). Unfortunately, at present, there is a paucity of epidemiological information on DEEs. To address this need, with the support of the national network of the Commission for Genetics of the Italian League Against Epilepsy (LICE), we conducted a survey of DEEs with an established molecular diagnosis over a 11-year period. These data were used as a proxy to estimate the prevalence rate of DEEs and to extrapolate epidemiological estimates. A quantitative estimate thus conducted is inevitably biased toward underestimation but provides at least a measure of the minimum number of affected individuals and the proportional distribution of specific genetic forms. Fifteen paediatric and adult tertiary Italian epilepsy centres participated in the survey. We also assessed how the odds of receiving a molecular diagnosis of DEE in Italy changed over the time interval under study.

## Methods

### Centres

We selected the 15 participating LICE centres based on several criteria, including geographic coverage across the nation (online supplemental table S1 and figure S1), high attractiveness for DEE, and available facilities to perform molecular genetic testing. Participating centres were Meyer Children’s Hospital IRCCS, Florence, Tuscany; Bambino Gesù Children’s Hospital, IRCCS, Rome, Lazio; Azienda Ospedaliera Universitaria Integrata di Verona, Verona, Veneto; IRCCS Eugenio Medea, Lecco, Lombardy; IRCCS Istituto Neurologico Carlo Besta, Milan, Lombardy; IRCCS Mondino Foundation, Pavia, Lombardy; IRCCS Istituto Giannina Gaslini, Genoa, Liguria; IRCCS Istituto delle Scienze Neurologiche di Bologna, Bologna, Emilia Romagna; Fondazione Policlinico Universitario Agostino Gemelli, IRCCS, Rome, Lazio; IRCCS Casa Sollievo della Sofferenza, San Giovanni Rotondo, Apulia; Azienda Ospedaliero-Universitaria Policlinico Umberto I/Sapienza Università di Roma, Rome, Lazio; Presidio Ospedaliero G. Salesi, Azienda Ospedaliero Universitaria delle Marche, Ancona, Marche; Associazione Oasi Maria SS. ONLUS—IRCCS, Troina, Sicily; Azienda Ospedaliera Universitaria Mater Domini, Catanzaro, Calabria; Azienda Ospedaliera Brotzu, Cagliari, Sardinia (online supplemental table S1).

### DEE gene selection

We established the list of EE/DEE genes to include in the survey based on a recent comprehensive overview of DEE^2^ and using the ‘developmental and epileptic encephalopathy’ phenotypic series as a query on the Online Mendelian Inheritance in Man (OMIM) database (https://omim.org/; MIM code: PS308350). After discussion among the participating centres, we integrated, modified and narrowed down the gene list to obtain a final list of 98 genes included in the molecular diagnostic workflows of most centres (online supplemental table S2). We did not include in the final list those genes identified after 2021 to avoid their prevalence to be underestimated.

### Patients’ cohort recruitment

We included in the survey patients who met the following criteria: (1) clinical diagnosis of DEE; (2) pathogenic or likely pathogenic variant(s) in one of the genes included in the survey; (3) molecular diagnosis established between 1 January 2012 and 3 December 2022.

We excluded patients if carrying pathogenic or likely pathogenic variants in more than one gene, that is, digenic or oligogenic phenotypes.

We gathered patients’ data in a template collecting: the identifier of the referring centre, the pseudonymisation code, the last 10 of the 16-digit tax ID code—including the birth’s year of the patient’s, the gene name related to the diagnosis and the year in which the diagnosis was made. We then calculated the age at molecular diagnosis and extrapolated gender information from the patients’ tax ID code.

Since some patients were likely to have been followed up in more than one centre across the country, we searched for duplicates by cross-checking the tax ID codes and ensure that everyone would be counted only once. We used the 10 digits of the ID code to retrieve the Italian region of birth by applying the ‘correlation’ tables supplied from the Italian National Institute of Statistics (ISTAT; http://www.istat.it). We identified patients born outside the Italian territory, either Italian citizens born abroad or foreigners, through the ‘Z’ letter followed by the state identification number (three digits) in the patients’ birthplace part of the tax ID code. We included these patients in the ‘Extra-Italy’ group. We obtained written informed consent from all participants or their legal guardians according to local requirements for genetic testing.

### Statistical analysis

We performed statistical analysis using Microsoft Excel and R V.4.0 (R Institute, Vienna, Austria). Once obtained the Italian geographical map (shapefile in the WGS84 reference system) by the Italian National Institute of Statistics (ISTAT; https://www.istat.it/it/archivio/222527), we used R V.4.0 to plot the survey’s data on the Italian map with regional boundaries. We obtained annual live birth data from the Italian National Institute of Statistics (ISTAT; http://dati.istat.it/) and normalised the number of patients with an identified molecular cause to the number of live births for the corresponding year, starting from 2000. We then calculated the incidence of molecular diagnoses of DEEs per live birth.

### Data availability

The authors affirm that all data necessary for confirming the conclusions of the article are present within the article, figures, tables and online supplemental material dataset (excluding patients’ sensitive information).

## Results

We collected 1825 patients’ records and discarded records with missing/largely incomplete tax ID code (n=58). We assessed the remaining 1767 records to rule out multiple entries for the same individual and found 1380 unique records and 354 records that had been submitted by two different centres, that is, 177 unique records, and 33 by three centres, that is, 11 unique records. After this redundancy check, we discarded 199 entries and included 1568 unique patients in the analysis. We assigned these patients to their birth’s region (n=1479) or to the ‘Extra-Italy’ group (n=89) (online supplemental table S3). Among the selected genes, we observed at least one record for 85 out of 98 genes. No causative variants were reported for *AARS1, CACNA1B, CUX2, KCNQ5, NACC1, NECAP1, PIGB, PLCB1, SIK1, SYNJ1, SLC25A12, UGP2* and *VARS1* (online supplemental table S2). The contribution of the 15 Italian centres to the survey is presented in online supplemental table S4.

### Survey quality control

**Figure 1:**
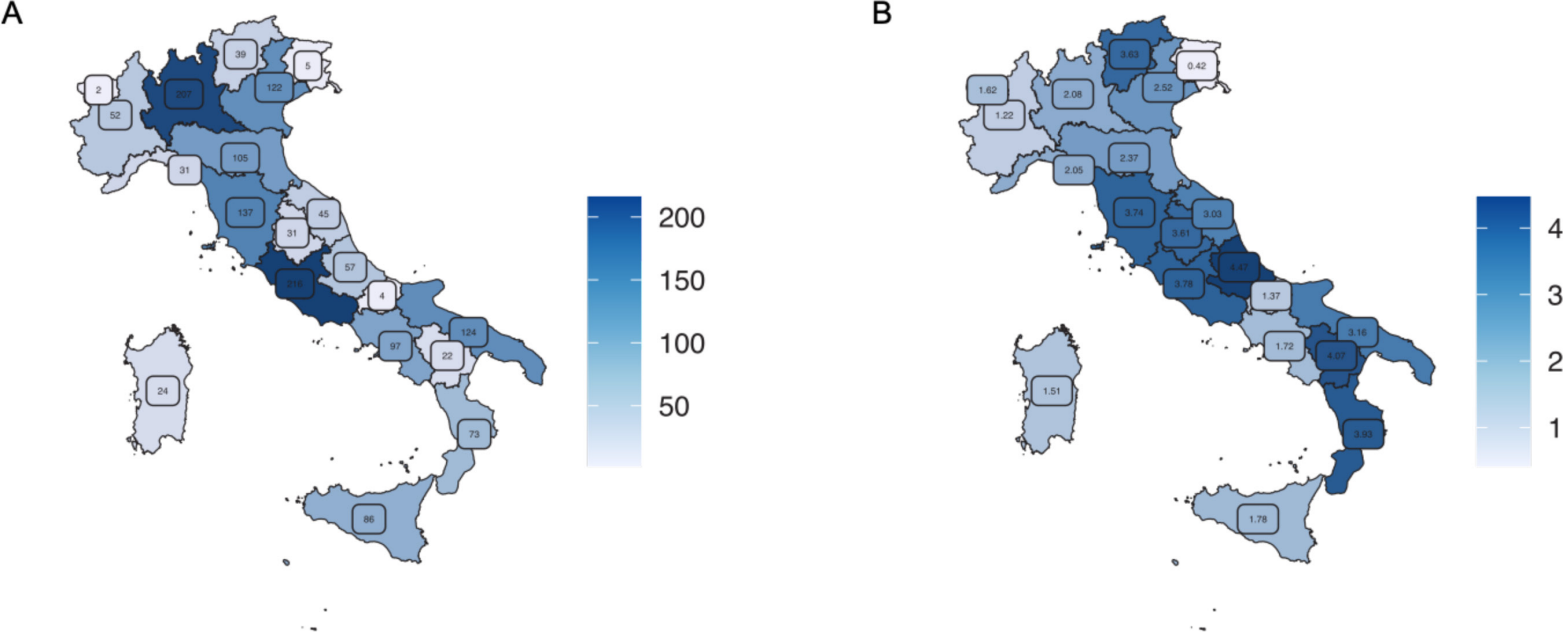
(**A**) Number of patients born in the 20 Italian regions and enrolled in the survey. (**B**) Mean incidence proportion of patients (2012–2022) per region normalised with the corresponding number of inhabitants. Similar blue intensities correspond to a similar mean incidence proportion of patients.

To prove our capability to identify patients with DEEs across the country, we assumed that the number of patients identified in each Italian region would be comparable after normalisation with the general population. To perform this calculation, we normalised the number of enrolled patients from the 20 Italian regions by the corresponding number of inhabitants on 1 January 2022 (ISTAT; http://dati.istat.it/) (figure 1A,B). We obtained a mean incidence proportion (2012–2022) of 2.6 patients for 100.000 inhabitants (SD=1.13) and observed consistent values across most Italian regions (online supplemental table S3) except for Friuli-Venezia Giulia where the incidence proportion was significantly lower, highlighting a possible under ascertainment of patients born in this region.

### Demographic characteristics

**Figure 2:**
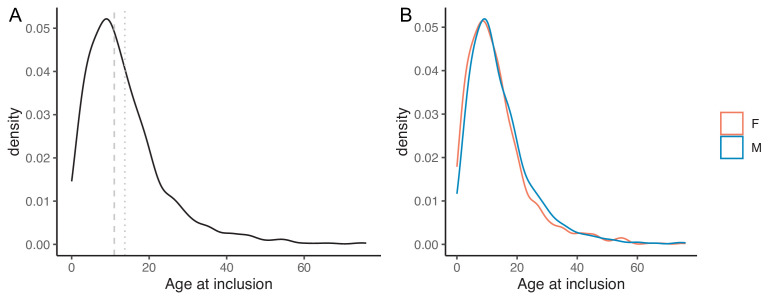
(**A**) Density plot of the age at the inclusion in the survey. (**B**) Density plot of the age at the inclusion in the survey grouped by sex. Vertical dashed grey line: median age (11 years); Vertical dotted grey line: mean age (13.7 years). Orange: female patients, light blue: male patients.

The survey cohort included 877 females and 691 males (1:1.27, M:F ratio). The mean age at the time of inclusion in the survey was 13.7 years (range from birth to 76 years—median 11 years). This is purely theoretical data since the study design did not allow us to know whether any demise had occurred. The density plot for age at inclusion is illustrated in figure 2A,B, including both the whole patient sample and patients grouped by sex.

### Number of DEE molecular diagnosis

**Figure 3:**
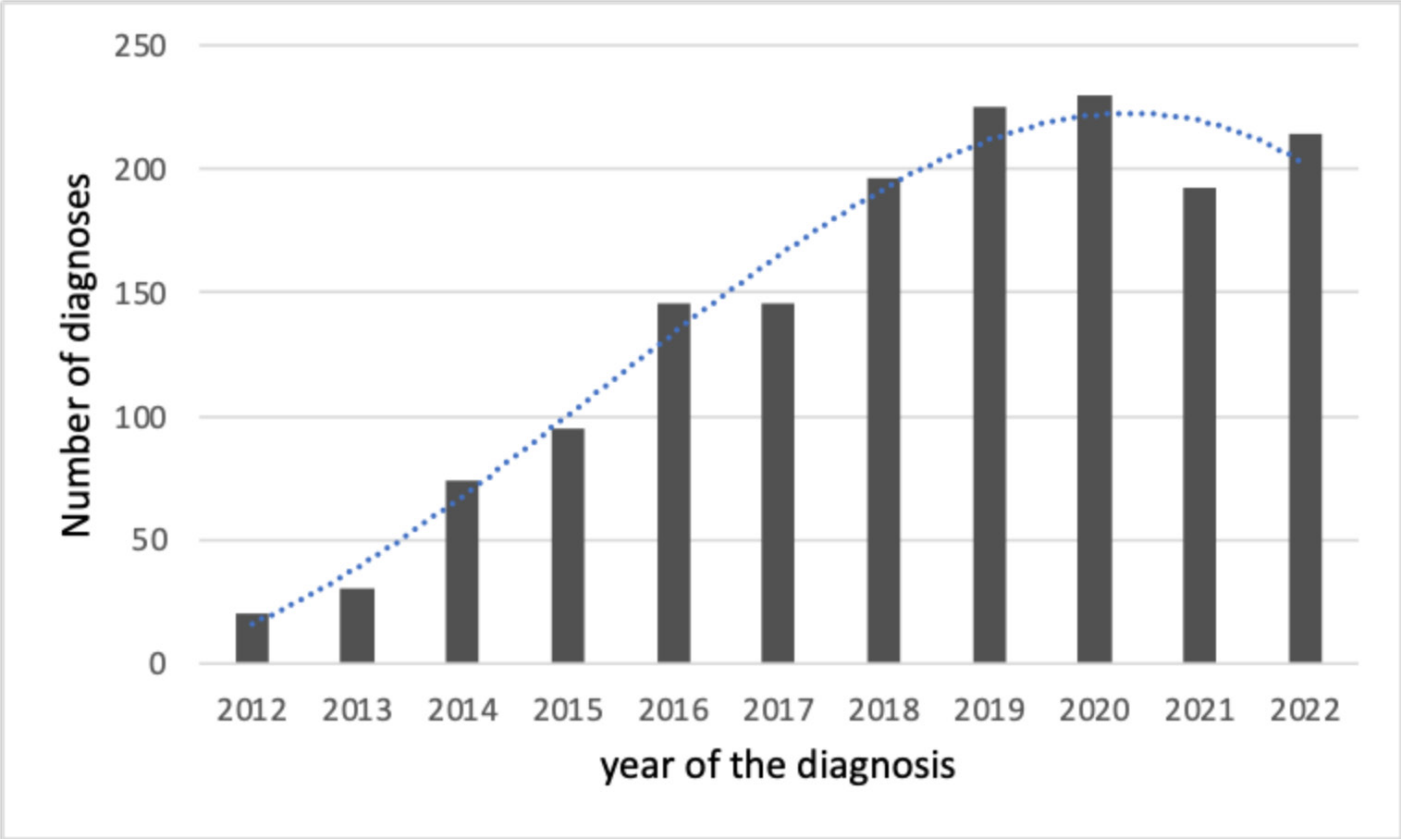
Per year distribution of the number of diagnoses reported in the survey. The dotted blue line depicts the polynomial (third order) trendline.

The number of molecular diagnoses obtained between 2012 and 2022 showed a continuing positive trend (figure 3), with 20 diagnoses in 2012 and 214 in 2022, thus resulting in more than a 10-fold increase. The incidence per 100 000 live births of patients with an identified molecular cause of DEE showed a tendency to plateau between the 2012 and 2020 birth years (online supplemental figure S2). The mean incidence of molecular diagnoses of DEEs during this period was 1 per 6277 live births (15.93/100 000; 95% CI 14.87 to 17.00).

### Age at molecular diagnosis

**Figure 4:**
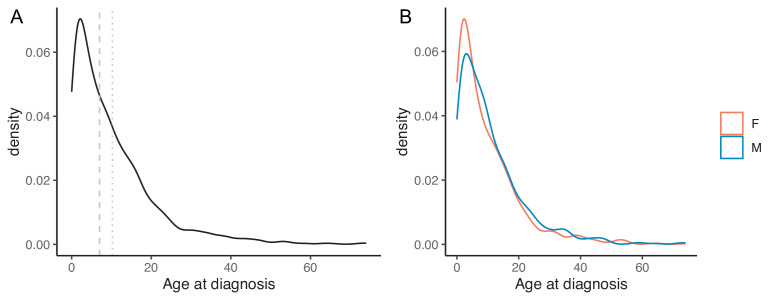
(**A**) Density plot of the age at molecular diagnosis. (**B**) Density plot of the age at molecular diagnosis grouped by sex. Vertical dashed grey line: median age (7 years); Vertical dotted grey line: mean age (10.2 years). Orange: female patients; light blue: male patients.

Mean age at molecular diagnosis was 10.2 years (median 7 years, range 0–74 years). The density plot of age at molecular diagnosis is illustrated in figure 4A,B, including both the whole patient sample and patients grouped by sex. Patients’ age at molecular diagnosis density plots, specific for each gene, were obtained for 85 out of 98 genes included in the survey (online supplemental figure S3). For 10/85 genes, patients’ age at molecular diagnosis density plots was empty since we observed only one patient per gene.

### Inheritance pattern

Pathogenic or likely pathogenic variants in genes with an autosomal dominant inheritance pattern occurred in 77% (n=1207) patients; 17% (n=271) in X-linked genes and 6% (n=90) in genes with autosomal recessive inheritance (online supplemental figure S4).

### Top-20 genes in the survey

The most frequently reported genes in the survey were *SCN1A* (16%), followed by the *KCNQ2* (5.5%) and *SCN2A* (5%). The top-20 genes are reported in online supplemental figure S5.

### Limitations

Our survey includes the main, but not all, Italian centres that follow patients with DEEs and laboratories that perform molecular analysis for these patients. Considering that the Italian healthcare system allows the free movement of patients throughout the country, it is very unlikely that a significant number of individuals with DEEs have escaped diagnosis in the highly specialised tertiary services for epilepsy participating to the survey and their related genetic laboratories. However, it must be considered that adults or elderly people with DEE residing in institutions or with limited prospects of clinical benefit from a late diagnosis have never been tested. Consequently, the number of patients with genetic DEEs is certainly underestimated, but the number of those who received an EE/DEE molecular diagnosis during the survey period is only minimally underestimated. We also acknowledge a risk of overestimation due to the lack of data on deceased patients with DEE. In a retrospective analysis of 510 individuals with genetic DEE, including pathogenic variants in *SCN1A, SCN2A, SCN8A, SYNGAP1, NEXMIF, CHD2, PCDH19, STXBP1, GRIN2A, KCNT1* and *KCNQ2*, and Angelman syndrome, Donnan *et al* reported 8% of deaths, producing a mortality rate of 6.1 per 1000 person-years (95% CI 4.4 to 8.3).^3^ These estimates can help correcting our prevalence figures, considering that all but one (ie, Angelman syndrome) genetic aetiologies were included in our survey.

The list of genes to be included in the survey was chosen after discussions among the participating centres and is certainly not inclusive of all genes associated with DEE. One of the selection criteria was that the genes had to be included in most diagnostic panels from different centres. These panels covered a range of conditions, including some that addressed also progressive diseases that can cause DEE, such as metabolic conditions, while others focused more on static DEEs. Lack of homogeneity in diagnostic coverage resulted in an unavoidable bias in the genes included in our survey.

Considering the 11-year survey period, it is unavoidable that the diagnostic strategies used in each centre to reach a genetic diagnosis were heterogeneous. Different sequencing techniques, including Sanger sequencing and next-generation sequencing (NGS) approaches such as small to large gene panels and whole exome sequencing, were used. In addition, small copy number variants (CNVs) involving one or a few exons could have been missed, as specific techniques (such as Multiplex Ligation-dependent Probe Amplification, MLPA) or bioinformatic algorithms were not available or included in the diagnostic workflows used in the different centres over the survey period.

Some of the genes included in the survey were not recognised as disease-causing genes at the beginning of the study, with a few only recently identified. Furthermore, the list of genes included in the survey does not encompass many additional genes known to cause DEE, even if only in a smaller subset of patients. These limitations could have influenced the results and the estimated prevalence of DEE.

## Discussion

Our country-wide survey on DEE has included a significant number of genes and identified a large population of patients with monogenic DEE. Its unblinded retrospective design and its extension to diagnoses made over 11 years imply that genetic testing strategies varied over time.

To better interpret the survey’s results, it is important to know that the Italian state has instituted a universal public healthcare system (the National Health System) since 1978. This system is highly decentralised, with each region being in charge of organising and delivering health services to the population, while each citizen remains free to move to where they feel better care is available.

We included in the analysis 1568 unique patients harbouring pathogenic or likely pathogenic variants in 85 out of the 98 selected genes (86.7%) (online supplemental table S2). The mean age at molecular diagnosis was 10.2 years (range: from 0 to 75 years, median: 7 years), highlighting the predominantly paediatric nature of the studied population. We observed 89 patients from the ‘Extra-Italy’ group, accounting for 5.6% of the cohort. This contingent of DEE derives from the immigrant population with a regular residence permit for long-term residence who are granted access to the national public healthcare system.

In the whole cohort, the autosomal dominant model was the most frequent (77%), whereas X-linked and autosomal recessive models were observed in 17% and 6% of patients. Both the autosomal dominant and the X-linked patterns were boosted by the large contribution of *de novo* variants that are identified in most DEE.^4^,^6^ Despite recessive variants in causative genes have been reported in 11–38% patients with DEE,^7 8^ we observed an autosomal recessive pattern of inheritance only in 6% of patients. This figure, although generated without the contribution of variants in genes following an X-linked recessive pattern, remains lower than expected. A possible explanation might derive from the low rate of consanguinity in the Italian population.^9^ In addition, considering that the interpretation of biallelic variants in autosomal recessive genes is more challenging compared with single variants in genes with dominant (mainly de novo) inheritance, it is possible that the number of likely pathogenic or pathogenic variants in autosomal recessive genes has been underestimated.

DEEs have a high genetic heterogeneity and the distribution of causative variants implies that only a few genes are linked to many cases while for most genes, only a few patients, are bound to each individual gene.^2^ The top-20 genes most frequently reported in this survey are consistently present at high diagnostic yield in retrospective studies in DEE,^10^,^13^ with *SCN1A, KCNQ2* and *SCN2A* being the top-3 as also reported by Heyne *et al*.^10^

Only a few studies have addressed the need for epidemiological data with prospective studies in genetic epilepsies. Symonds *et al* reported on the incidence of the most common single-gene epilepsies in a prospective population-based national cohort recruited in Scotland over a 3-year period.^14^ These authors performed genetic testing through a custom-designed 104-gene epilepsy panel in 333 children presenting with seizures before 36 months of age. Of these, 80/333 (24%) had a diagnostic genetic finding, and 27/80 (33.8%) were DEE. The overall estimated annual incidence of single-gene epilepsies in this population was 1 per 2120 live births (47.2/100 000; 95% CI 36.9 to 57.5), with pathogenic variants in *SCN1A, PCDH19, CDKL5* and *KCNQ2,* having the highest incidence for DEE. In our survey, the mean incidence of molecular diagnoses of single-gene DEEs among live births in the 2012–2020 time frame was 1 per 6277 (15.93/100 000; 95% CI 14.87 to 17.00). This figure provides a mean incidence of single-gene DEEs lower than that estimated by Symonds *et al* (1 per 2120 live births). Differences in experimental design (prospective vs retrospective) and inclusion criteria may account for the different estimates.

The same group (Symonds *et al*) provided a further epidemiological characterisation of early-onset epilepsies in the Scottish population through a 3-year prospective recruitment strategy also using an independent case ascertainment strategy. Of the 390 children included, 146 had a DEE (37.4%). The incidence of all DEEs was 86.1 per 100 000 live births (95% CI 72.7 to 101.3). Among DEEs, the highest incidence was for infantile spasms syndrome, 30.7 per 100 000 live births (95% CI 22.9 to 40.2) [genetic aetiologies identified in 22/52 (42%), including trisomy 21 (n=6), and pathogenic variants in *CDKL5* (n=2)*, TSC1* (n=2)*, TSC2* (n=3)], followed by early infantile DEE (<3 months), 10 per 100 000 live births (95% CI 5.8 to 16.0) [genetic aetiologies identified in 12/17 (70.5%), including pathogenic variants in *CDKL5 (n=2*)], and Dravet syndrome, 6.5 per 100 000 live births (95% CI 3.2 to 10.0), caused by *SCN1A* pathogenic variants in all individuals (n=11). Other DEEs without a specific syndromic classification had an incidence of 31.9 per 100 000 live births (95% CI 23.9 to 41.6), with a genetic aetiology identified in 19/54 (35%) including 15q11-13 deletion (Angelman, n=2), 16p11.2 deletion (n=2), pathogenic variants in *PCDH19 (n=2*) and *SLC6A1* (n=2).^15^ It is difficult to compare our findings with those generated by population-based studies due to differences in methodology. In addition, we included DEE phenotypes without specific syndromic characterisation but with an established genetic aetiology and did not consider chromosomal abnormalities. However, the most prevalent single gene aetiologies emerging from our survey are concordant with those reported by Symonds *et al*, especially for *SCN1A-* and *CDKL5*-related DEEs.^14 15^

Our survey provides an imperfect but reliable quantitative estimate on the set of rare diseases grouped under the definition of DEEs. This information is of paramount importance together with knowledge about the functional alterations underlying a given genetic DEE to formulate plans for approaching precision therapies. A precision medicine approach implies capacity and intention to switch from medications treating seizures at large to molecules tailored towards specific conditions manifesting seizures as one of the symptoms, a paradigm shift with considerable marketing implications.^16^ In this perspective, we tried to measure the magnitude of DEE patients’ population and their specific single gene causes in a large European country, thus providing a dataset to be used for decision-making strategies both for pharmaceutical purposes and regulatory agencies. Our study also provides information about the least rare DEEs among rare DEEs, thus highlighting interests in the development of specific molecules for treatment.

The number of molecular diagnoses collected in the survey showed a continuing positive trend in the number of diagnoses over time, with about a 10-fold increase over the 11-year time frame. This was likely determined by the growth in the number of genes associated with epileptic encephalopathies,^2 17^ and the increased use of wide NGS-targeted gene panels and exomes in clinical practice.^13 18^ The slight slowdown of the molecular diagnoses in the last three reference years of the survey (2020–2022) can be ascribed in part to the COVID-19 pandemic and, for the last year (2022), the incomplete receipt from genetics laboratories of all test results initiated that year. Looking at the whole data trend, we do not expect an increase of molecular diagnoses as substantial as in the past. Yet a constant increase remains likely since omics technologies such as the long reads sequencing, the optical genome mapping and the improved knowledge related to the interpretation of variants in non-coding regions of the human genome will likely result in increasing the DEE with a molecular diagnosis.^16^

Some geographical/regional differences in the distribution of patients with DEE emerged after normalisation with the number of inhabitants. In particular, the observed mean incidence proportion was significantly lower in the Friuli-Venezia-Giulia region (0.42 for 100.000 inhabitants) in comparison to the country-wide mean of 2.6 for 100.000 inhabitants. This five-fold lower value can be ascribed to an under ascertainment of the patients with DEE from this region, possibly due to the absence of a local centre in the survey. Considering the country-wide patients mean incidence proportion, we also observed slightly lower values in some regions for which a local centre was not included in the survey (ie, Piemonte, Molise and Campania).

The estimate of DEEs provided in this study does not include those due to chromosomopathies, large CNVs, mitochondrial DNA variants, nor all undiagnosed cases. The purpose of our study, however, was to provide even a rough estimate of the forms related to potentially actionable monogenic mechanisms, not to make an estimate of all patients with DEEs present in Italy.

Although our survey likely underestimates the total number of patients who received a DEE molecular diagnosis in Italy, it provides an analytical estimate that is essential for informing decision-makers in drug development on the appropriateness of initiatives aimed at developing precision medicine therapies. Knowledge of the magnitude of affected populations with specific genetic conditions underlying DEEs is also instrumental to implement disease-specific registries^19^ and facilitate natural history studies.

## supplementary material

10.1136/jmg-2024-110328 online supplemental file 1

10.1136/jmg-2024-110328 online supplemental file 2

## Data Availability

All data relevant to the study are included in the article or uploaded as supplementary information.
