# Supplementary material for: National survey on the prevalence of single-gene aetiologies for genetic developmental and epileptic encephalopathies in Italy
Source: J Med Genet. 2024 Nov 28;62(1):e110328. doi: 10.1136/jmg-2024-110328 (PMC11877070; doi:10.1136/jmg-2024-110328)
Supplement: online supplemental file 1 [file jmg-62-1-s001.pdf]

Supplemental Material

A National Survey on the Prevalence of Single-Gene Aetiologies for Genetic Developmental and Epileptic Encephalopathies in Italy

Centre Name	City	Region	Type of centre
Meyer Children's Hospital IRCCS	Florence	Tuscany	Clinical and Molecular
Bambino Gesù Children's Hospital, IRCCS	Rome	Lazio	Clinical and Molecular
Azienda Ospedaliero-Universitaria Integrata Verona	Verona	Veneto	Clinical
Scientific Institute IRCCS E. Medea	Bosisio Parini	Lombardy	Clinical and Molecular
Fondazione IRCCS Istituto Neurologico Carlo Besta	Milan	Lombardy	Clinical and Molecular
IRCCS Mondino Foundation	Pavia	Lombardy	Clinical and Molecular
IRCCS Istituto Giannina Gaslini	Genoa	Liguria	Clinical and Molecular
IRCCS Istituto delle Scienze Neurologiche di Bologna	Bologna	Emilia Romagna	Clinical and Molecular
Fondazione Policlinico Universitario Agostino Gemelli, IRCCS	Rome	Lazio	Clinical
Fondazione IRCCS Casa Sollievo della Sofferenza	San Giovanni Rotondo	Apulia	Clinical and Molecular
Azienda Ospedaliero-Universitaria Policlinico Umberto I / Sapienza Università di Roma	Rome	Lazio	Clinical
Presidio Ospedaliero G. Salesi, Azienda Ospedaliero Universitaria delle Marche	Ancona	Marche	Clinical
Associazione Oasi Maria SS. ONLUS – IRCCS	Troina	Sicily	Clinical and Molecular
Azienda Ospedaliera Universitaria Mater Domini	Catanzaro	Calabria	Clinical and Molecular
ARNAS G. Brotzu	Cagliari	Sardinia	Clinical and Molecular

Table S1. List of the 15 centres participating to the survey.

Gene name	Inheritance (according MIM)	Gene/Locus MIM number	Cytoband	Pts n°
AARS1	AR	601065	16q22.1	0
ALG13	XL	300776	Xq23	6
ARHGEF9	XL	300429	Xq11.1	4
ARV1	AR	611647	1q42.2	1
ARX	XL	300382	Xp21.3	7
ATP1A2	AD	182340	1q23.2	10
ATP1A3	AD	182350	19q13.2	13
ATP6V1A	AD	607027	3q13.31	3
BRAT1	AR	614506	7p22.3	10
CACNA1A	AD	601011	19p13.13	35
CACNA1B	AR	601012	9q34.3	0
CACNA1E	AD	601013	1q25.3	6
CAD	AR	114010	2p23.3	2
CASK	XL	300172	Xp11.4	15
CDK19	AD	614720	6q21	1
CDKL5	XL	300203	Xp22.13	61
CHD2	AD	602119	15q26.1	42
CLCN4	XL	302910	Xp22.2	3
CLTC	AD	118955	17q23.1	4
CSNK2B	AD	115441	6p21.33	19
CUX2	AD	610648	12q24.11-q24.12	0
CYFIP2	AD	606323	5q33.3	3
DHDDS	AD	608172	1p36.11	3
DNM1	AD, AR	602377	9q34.11	8
DNM1L	AD, AR	603850	12p11.21	3
DOCK7	AR	615730	1p31.3	1
EEF1A2	AD	602959	20q13.33	10
FGF12	AD	601513	3q28-q29	2
FOXG1	AD	164874	4q12	19
FRRS1L	AR	604574	9q31.3	2
GABRA1	AD	137160	5q34	27
GABRA5	AD	137142	15q12	3
GABRB1	AD	137190	4p12	1
GABRB2	AD	600232	5q34	5
GABRB3	AD	137192	15q12	22
GABRG2	AD	137164	5q34	29
GNAO1	AD	139311	16q13	22
GRIN1	AD, AR	138249	9q34.3	14
GRIN2A	AD	138253	16p13.2	49
GRIN2B	AD	138252	12p13.1	18
GRIN2D	AD	602717	19q13.33	2
HCN1	AD	602780	5p12	8
HNRNPU	AD	602869	1q44	5
KCNA2	AD	KCNA2	1p13.3	14
KCNB1	AD	KCNB1	20q13.13	23
KCNQ2	AD	602235	20q13.33	88
KCNQ5	AD	607357	6q13	0
KCNT1	AD	608167	9q34.3	39
KMT2E	AD	608444	7q22.3	8

Gene name	Inheritance (according MIM)	Gene/Locus MIM number	Cytoband	Pts n°
MBD5	AD	611472	2q23.1	7
MECP2	XL	300005	Xq28	71
MEF2C	AD	301002	5q14.3	5
NACC1	AD	610672	19p13.13	0
NECAP1	AR	611623	12p13.31	0
NEXMIF	XL	300524	Xq13.3	17
NTRK2	AD	600456	9q21.33	1
PACS2	AD	610423	14q32.33	5
PARS2	AR	612036	1p32.3	3
PCDH19	XL	300460	Xq22.1	62
PIGA	XL	311770	Xp22.2	8
PIGB	AR	604122	15q21.3	0
PIGQ	AR	605754	16p13.3	1
PLCB1	AR	607120	20p12.3	0
PNKP	AR	605610	19q13.33	10
PNPO	AR	610090	17q21.32	5
POLG	AR	174763	15q26.1	5
PURA	AD	600473	5q31.3	15
RHOBTB2	AD	607352	8p21.3	1
RNF13	AD	609247	3q25.1	1
SCN1A	AD	182389	2q24.3	252
SCN1B	AD, AR	600235	19q13.11	13
SCN2A	AD	182390	2q24.3	78
SCN3A	AD	182391	2q24.3	4
SCN8A	AD	600702	12q13.13	60
SIK1	AD	605705	21q22.3	0
SYNJ1	AR	604297	21q22.11	0
SLC12A5	AR	606726	20q13.12	2
SLC13A5	AR	608305	17p13.1	10
SLC1A2	AD	600300	11p13	2
SLC25A12	AR	603667	2q31.1	0
SLC25A22	AR	609302	11p15.5	1
SLC2A1	AD, AR	138140	1p34.2	49
SLC35A2	XL	300896	Xp11.23	3
SLC6A1	AD	137165	3p25.3	39
SLC9A6	XL	300231	Xq26.3	7
SMC1A	XL	300040	Xp11.22	7
SPTAN1	AD	182810	9q34.11	18
ST3GAL3	AR	606494	1p34.1	1
ST3GAL5	AR	604402	2p11.2	3
STXBP1	AD, AR	602926	9q34.11	53
SYNGAP1	AD	603384	6p21.32	41
SZT2	AR	615463	1p34.2	10
TBC1D24	AR	613577	16p13.3	12
UBA5	AR	610552	3q22.1	4
UGP2	AR	191760	2p15	0
VARS1	AR	192150	6p21.33	0
WWOX	AR	605131	16q23.1-q23.2	7
YWHAG	AD	605356	7q11.23	5

Table S2. List of the 98 genes included in the survey and the number of patients with (likely)-pathogenic variants in each gene. Genes in light grey were included in the survey but no centres reported patients(n°=13). AD: autosomal dominant; AR: autosomal recessive; XL: X-linked. Gene/Locus MIM number: Gene/Locus number as reported in www.omim.org. Pts n°: number of patients with (likely)-pathogenic variants in each gene.

Region Name	Patients	Populations (2022)	Mean incidence proportion / 100K
Abruzzo	57	1275950	4.47
Basilicata	22	541168	4.07
Calabria	73	1855454	3.93
Campania	97	5624420	1.72
Emilia Romagna	105	4425366	2.37
Friuli Venezia Giulia	5	1194647	0.42
Lazio	216	5714882	3.78
Liguria	31	1509227	2.05
Lombardy	207	9943004	2.08
Marche	45	1487150	3.03
Molise	4	292150	1.37
Piemonte	52	4256350	1.22
Apulia	124	3922941	3.16
Sardinia	24	1587413	1.51
Sicily	86	4833329	1.78
Tuscany	137	3663191	3.74
Trentino Alto Adige	39	1073574	3.63
Umbria	31	858812	3.61
Valle D'Aosta	2	123360	1.62
Veneto	122	4847745	2.52
**Total Italy**	**1479**	**59030133**	**2.51**
**Extra-Italy**	**89**	**-**	
**Total Survey**	**1568**		

Table S3. Patients enrolled in the survey assigned to their birth's region and the mean incidence proportion calculated on the basis of the 2022 Italian regional population.

Centre Name	Patients Enrolled
Meyer Children's Hospital IRCCS	575
Bambino Gesù Children's Hospital, IRCCS	237
Azienda Ospedaliero-Universitaria Integrata Verona	129
Scientific Institute IRCCS E. Medea	91
Fondazione IRCCS Istituto Neurologico Carlo Besta	74
IRCCS Mondino Foundation	73
IRCCS Istituto Giannina Gaslini	61
IRCCS Istituto delle Scienze Neurologiche di Bologna	60
Fondazione Policlinico Universitario Agostino Gemelli, IRCCS	60
Fondazione IRCCS Casa Sollievo della Sofferenza	59
Azienda Ospedaliero-Universitaria Policlinico Umberto I / Sapienza Università di Roma	54
Presidio Ospedaliero G. Salesi, Azienda Ospedaliero Universitaria delle Marche	44
Associazione Oasi Maria SS. ONLUS – IRCCS	19
Azienda Ospedaliera Universitaria Mater Domini	16
ARNAS G. Brotzu	16
**Total Survey**	**1568**

Table S4. Patients contributed from the 15 centres participating to the survey.

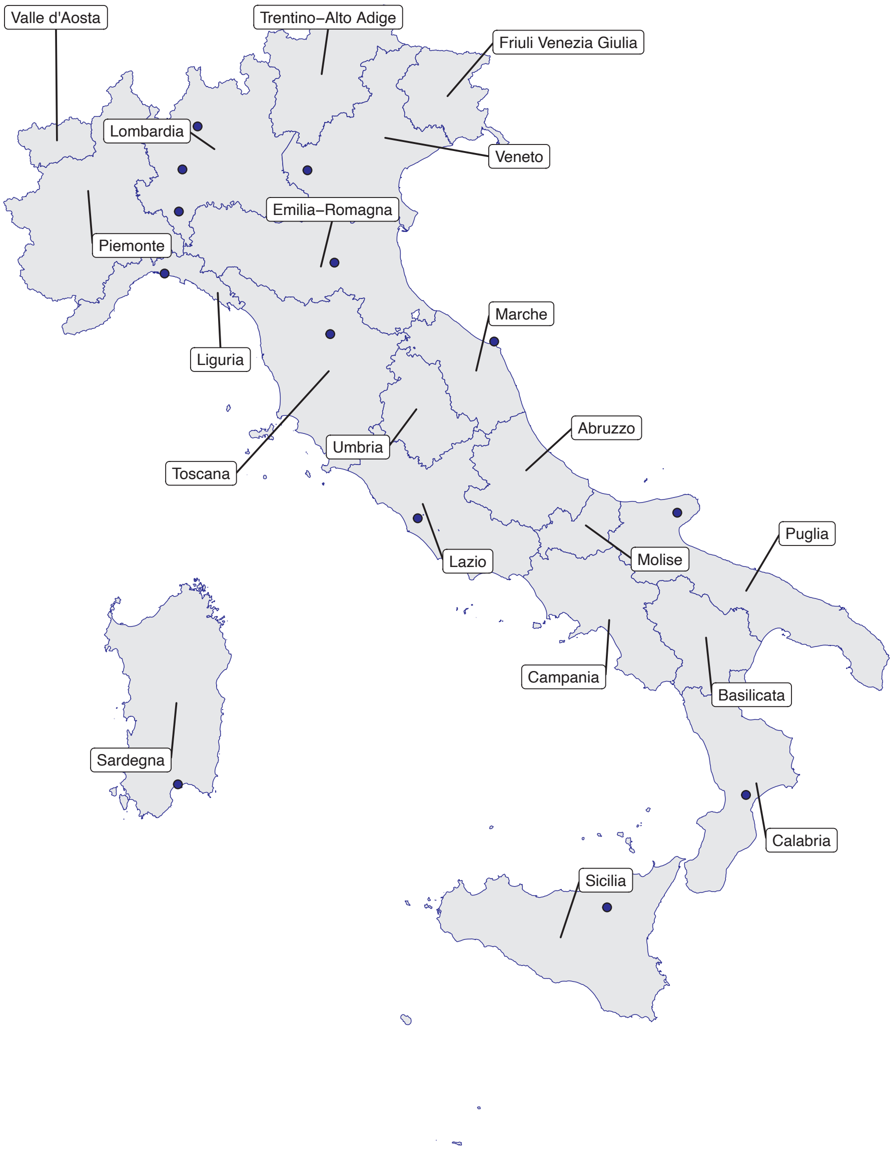

Figure S1. Geographical distribution of the 15 centres (black dots) involved in the survey. Rome has three centres (visible as a single black dot).

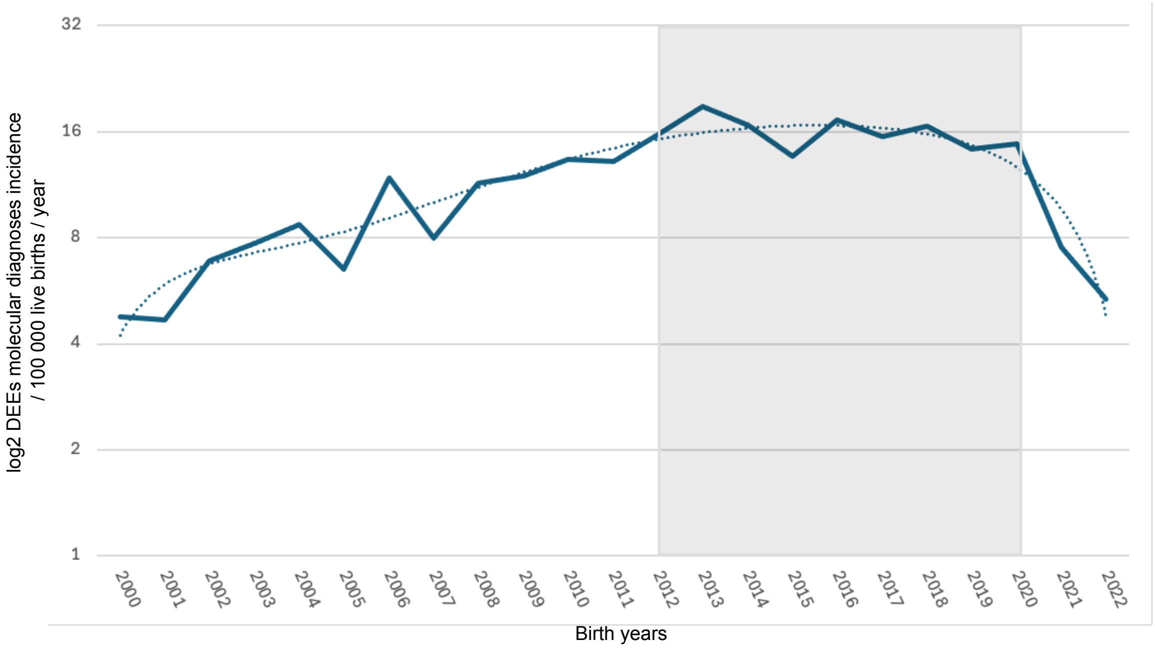

Figure S2. Semi-logarithmic plot of the DEEs molecular diagnoses incidence / 100 000 live births / year. The blue dotted polynomial trend line shows a tendency to plateau for the incidence in the 2012-2020 time frame (shaded in gray), within which we observed the molecular diagnoses of single-gene DEEs mean incidence to be 1 per 6277 live births (15.93/100 000; 95% CI 14.87-17.00).

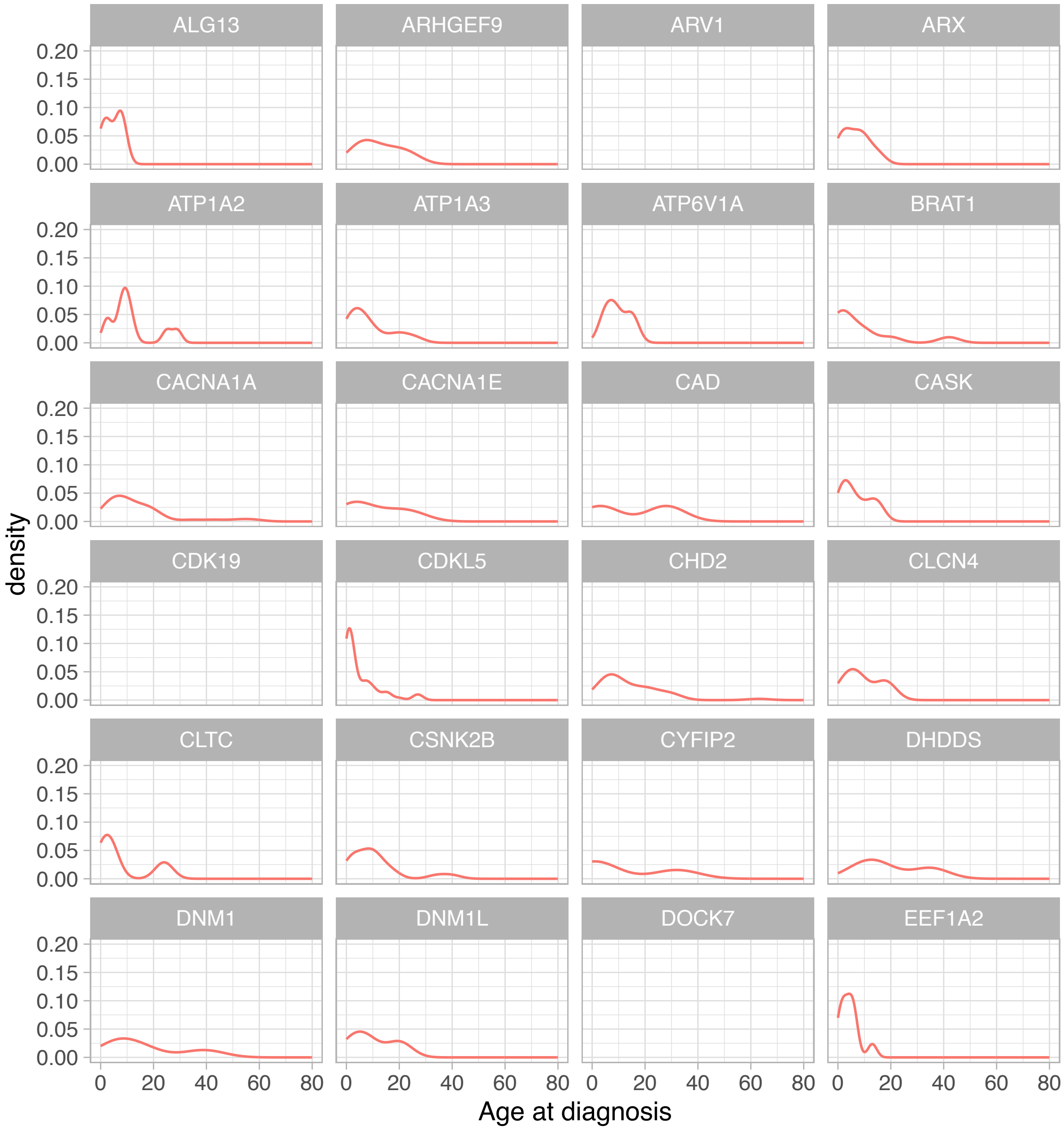

ALG13
ARHGEF9
ARV1
ARX
ATP1A2
ATP1A3
ATP6V1A
BRAT1
CACNA1A
CACNA1E
CAD
CASK
CDK19
CDKL5
CHD2
CLCN4
CLTC
CSNK2B
CYFIP2
DHDDS
DNM1
DNM1L
DOCK7
EEF1A2
density
0.00
0.05
0.10
0.15
0.20
0
20
40
60
80
Age at diagnosis

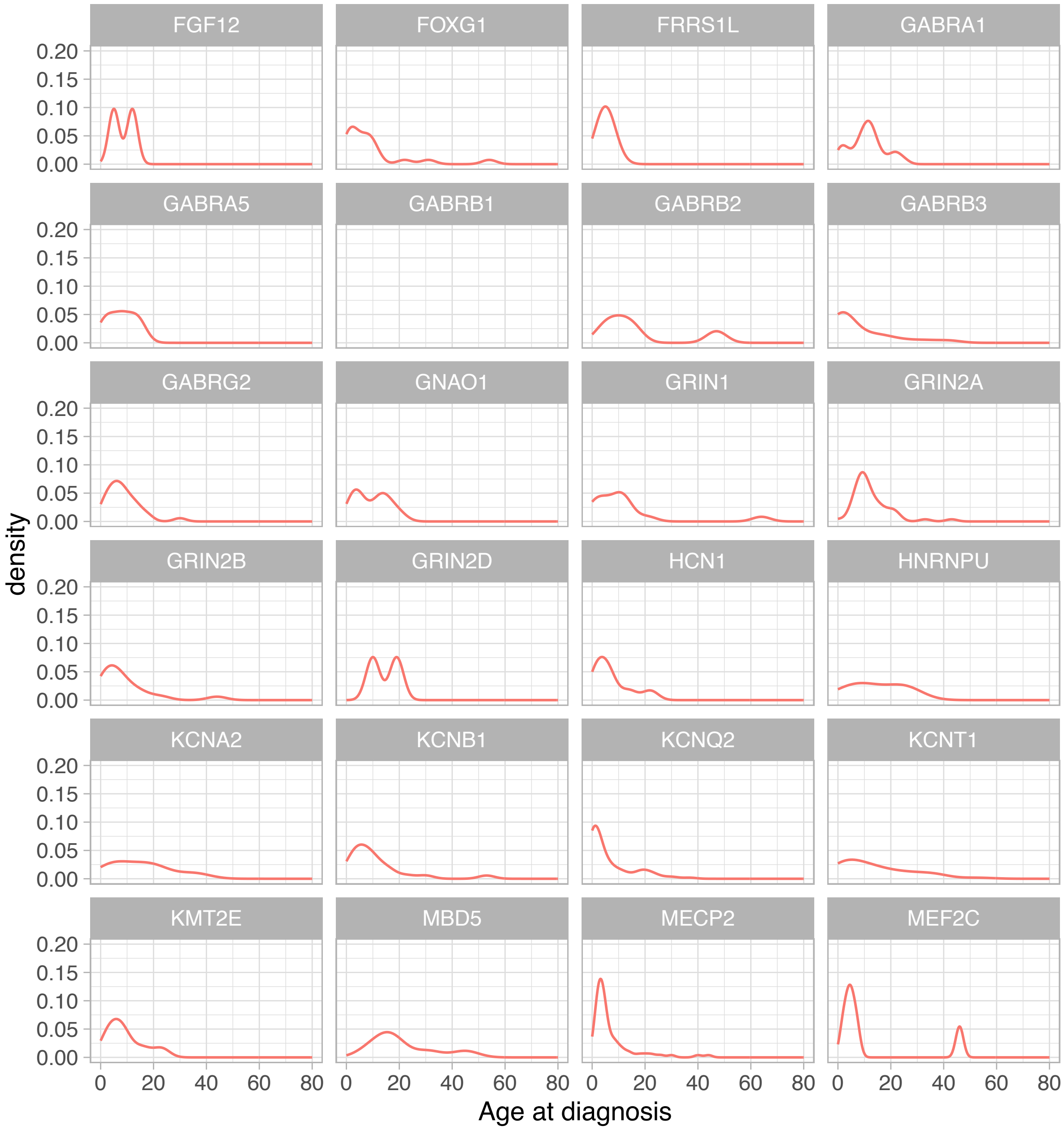
FGF12
FOXG1
FRRS1L
GABRA1
GABRA5
GABRB1
GABRB2
GABRB3
GABRG2
GNAO1
GRIN1
GRIN2A
GRIN2B
GRIN2D
HCN1
HNRNPU
KCNA2
KCNB1
KCNQ2
KCNT1
KMT2E
MBD5
MECP2
MEF2C
density
0.00
0.05
0.10
0.15
0.20
0
20
40
60
80
Age at diagnosis

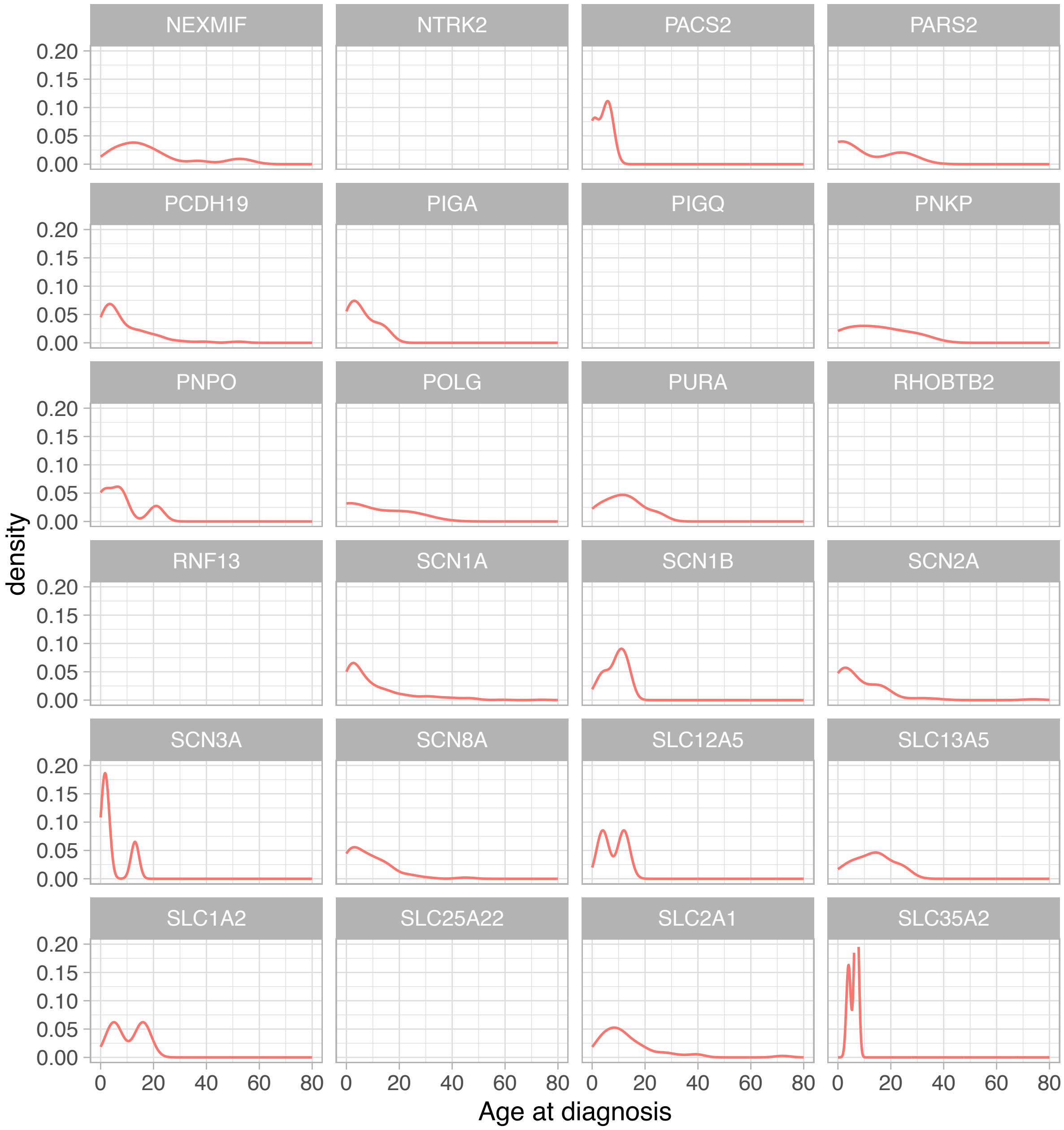

NEXMIF
NTRK2
PACS2
PARS2
PCDH19
PIGA
PIGQ
PNKP
PNPO
POLG
PURA
RHOBTB2
RNF13
SCN1A
SCN1B
SCN2A
SCN3A
SCN8A
SLC12A5
SLC13A5
SLC1A2
SLC25A22
SLC2A1
SLC35A2
0.00
0.05
0.10
0.15
0.20
0
20
40
60
80
density
Age at diagnosis

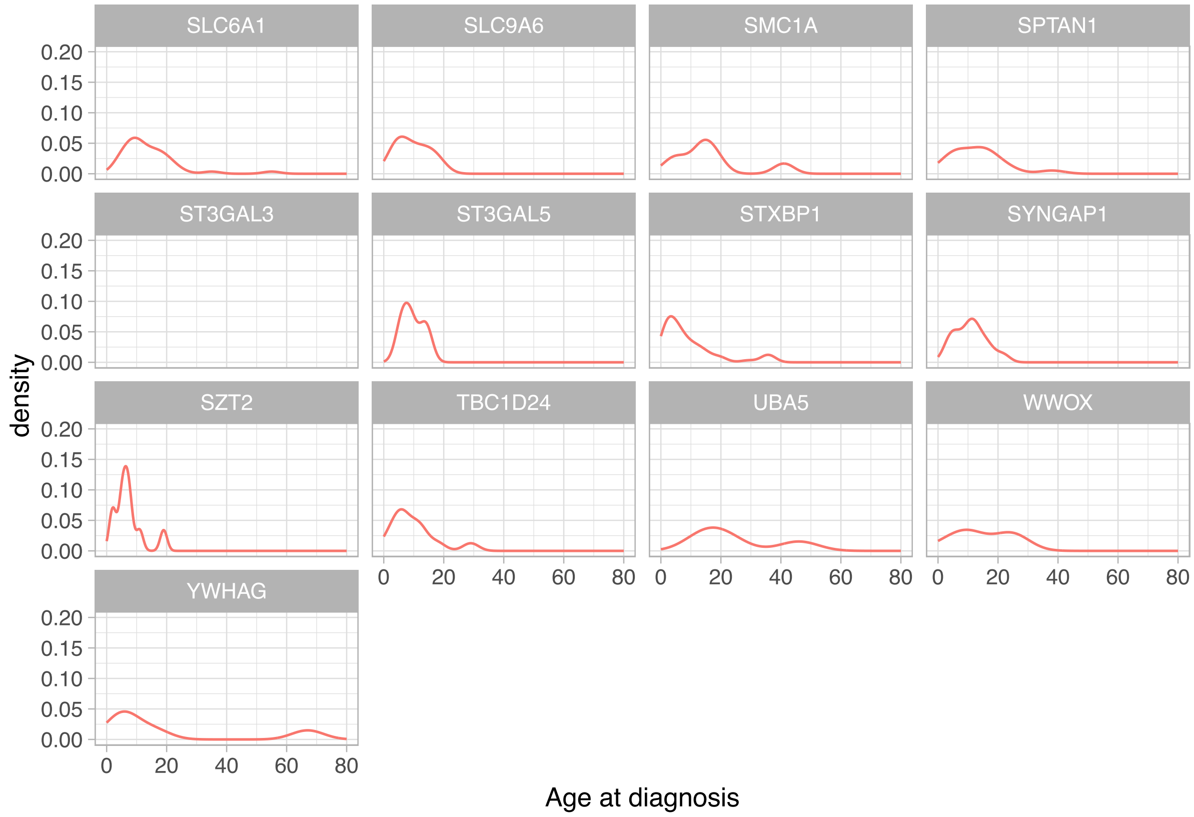

Figure S3. Age at molecular diagnosis density plots of the 85 genes for whom patient's records were available. For ten genes (*ARV1, CDK19, DOCK7, GABRB1, NTRK2, PIGQ, RHOBTB2, RNF13, SLC25A22, ST3GAL3*) the density plot is empty (patient's records < 2).

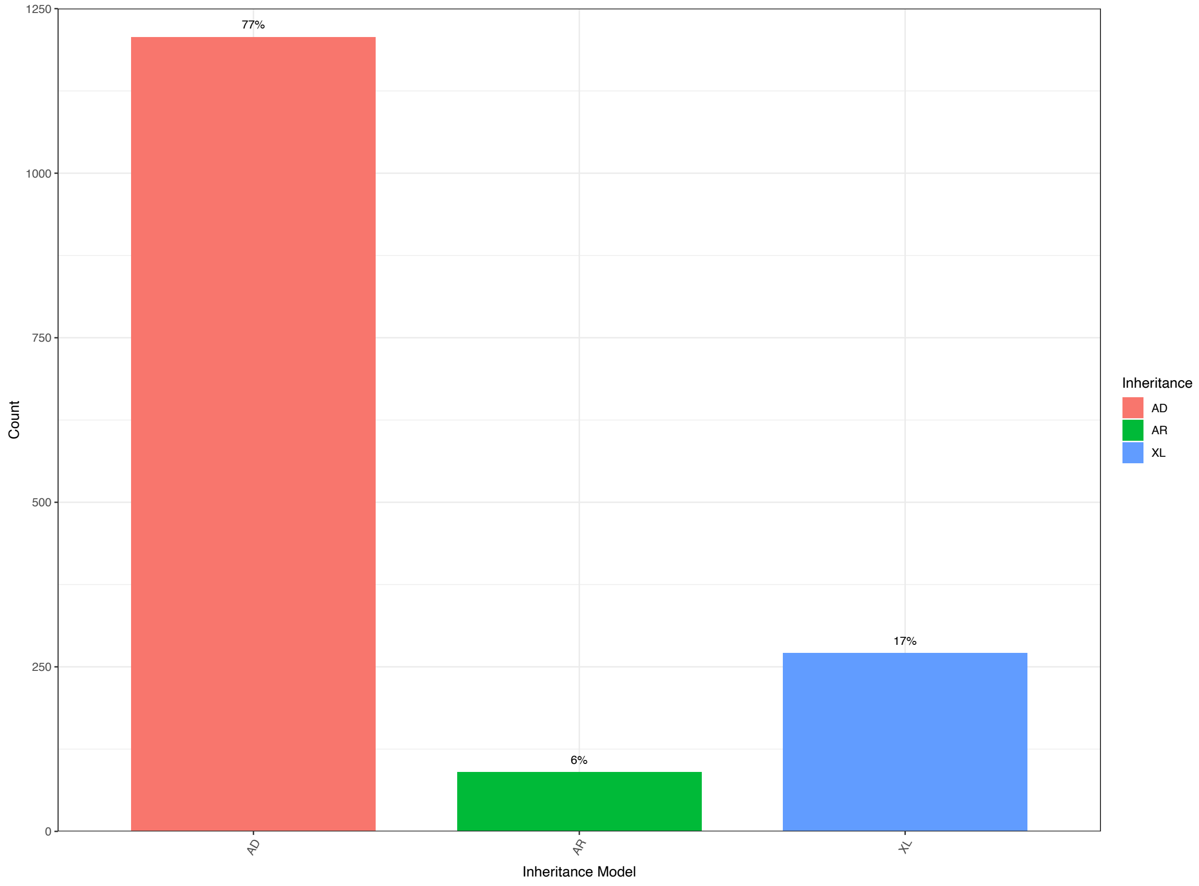

Figure S4. Bar plot of the observed inheritance models. Autosomal Dominant (AD), Autosomal Recessive (AR) and X-Linked (XL) inheritance patterns were observed in 77% (n=1207), in 6% (n=90) and in 17% (n=271) of the patients, respectively.

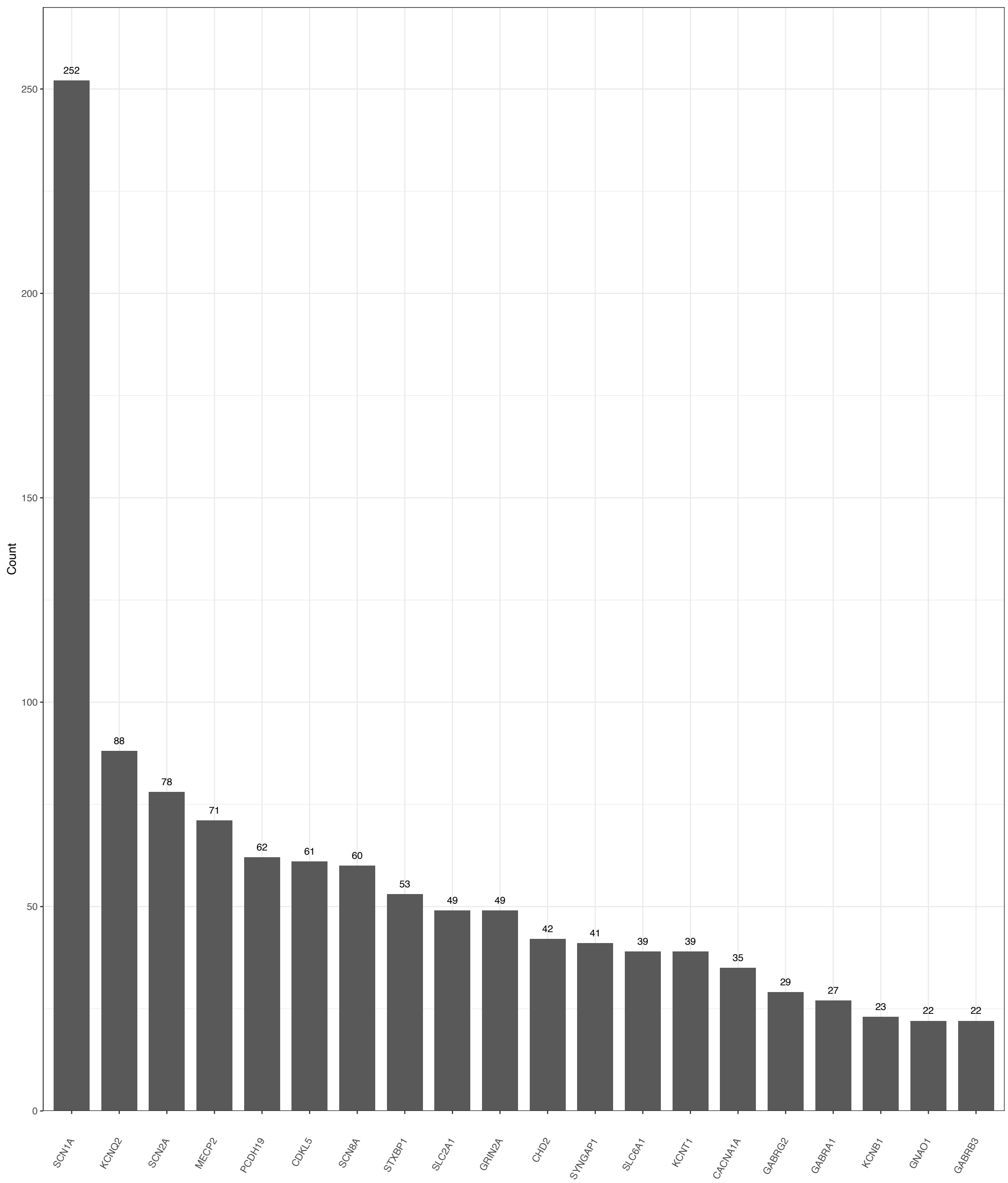

Figure S5. Top-20 most frequently reported genes in the survey.
