# Supplementary material for: National survey on the prevalence of single-gene aetiologies for genetic developmental and epileptic encephalopathies in Italy
Source: J Med Genet. 2024 Nov 28;62(1):e110328. doi: 10.1136/jmg-2024-110328 (PMC11877070; doi:10.1136/jmg-2024-110328)
Supplement: online supplemental file 2 [file jmg-62-1-s002.pdf]

Description of the data and file structure

We aimed to estimate real-world-evidence of the prevalence rate of genetic developmental and epileptic encephalopathies (DEEs) in the Italian population over a 11-year period.

Fifteen pediatric and adult tertiary Italian epilepsy centres participated to a survey related to 98 genes included in the molecular diagnostic workflows of most centers. We included in the survey patients with a clinical diagnosis of DEE, caused by a pathogenic or likely pathogenic variant in one of the selected genes, with a molecular diagnosis established between 2012 and 2022.

Description:

This is the dataset including data from the 1568 patients enrolled in the survey.

Variables

ID: Pseudoanonimyzation code

Referring_centre: The centre that has referred the patient(s). Each abbreviation correspond to a centre according the following list:

Bellaria-BO: IRCCS Istituto delle Scienze Neurologiche di Bologna, Bologna, Emilia Romagna
Besta-MI: IRCCS Istituto Neurologico Carlo Besta, Milan, Lombardy
Gaslini-GE: IRCCS Istituto Giannina Gaslini, Genoa, Liguria
Gemelli-RM: Fondazione Policlinico Universitario Agostino Gemelli, IRCCS, Rome, Lazio
IRCCSMEDEALC: IRCCS Eugenio Medea, Lecco, Lombardy
MaterDomini-CZ: Azienda Ospedaliera Universitaria Mater Domini, Catanzaro, Calabria
Meyer-FI: Meyer Children's Hospital IRCCS, Florence, Tuscany
Mondino-Pavia: IRCCS Mondino Foundation, Pavia, Lombardy
NeuroPedAOB: Azienda Ospedaliera Brotzu, Cagliari, Sardinia
NPIv.Sabelli-RM: Azienda Ospedaliero-Universitaria Policlinico Umberto I / Sapienza Università di Roma, Rome, Lazio
Oasi-Troina: Associazione Oasi Maria SS. ONLUS – IRCCS, Troina, Sicily
OBG-RM: Bambino Gesù Children's Hospital, IRCCS, Rome, Lazio
Salesi-Ancona: Presidio Ospedaliero G. Salesi, Azienda Ospedaliero Universitaria delle Marche, Ancona, Marche
SollievoeSoff.-FG: IRCCS Casa Sollievo della Sofferenza, San Giovanni Rotondo, Apulia
UOCNeuropsichiatriaInfantileVerona: Azienda Ospedaliera Universitaria Integrata di Verona, Verona, Veneto

Sex: the biological sex of the patient

Gene: the HUGO gene name involved in the molecular diagnosis of the patient

Age_at_molecular_diagnosis: Patient's age at molecular diagnosis

Age_at_the_time_of_inclusion: Patient's age at the time of inclusion (2022)

Italy_Region: Italian Region where the patient is born. "." stand for Extra-Italy

ID	Referring_centre	Sex	Gene	Age_at_molecular_diagnosis	Age_at_the_time_of_inclusion	Italy_Region
1	Bellaria-BO	F	SLC2A1	39	45	EMILIA ROMAGNA
2	Bellaria-BO	M	SCN1A	48	53	EMILIA ROMAGNA
3	Bellaria-BO	F	SCN1A	38	44	SICILIA
4	Bellaria-BO	F	PCDH19	7	13	CALABRIA
5	Bellaria-BO	F	SCN2A	6	12	EMILIA ROMAGNA
6	Bellaria-BO	F	SCN1A	32	37	.
7	Bellaria-BO	M	PCDH19	27	32	EMILIA ROMAGNA
8	Bellaria-BO	F	KCNQ2	1	5	EMILIA ROMAGNA
9	Bellaria-BO	F	GRIN2A	9	13	EMILIA ROMAGNA
10	Bellaria-BO	M	CACNA1A	21	25	EMILIA ROMAGNA
11	Bellaria-BO	M	SCN1A	3	7	PUGLIE
12	Bellaria-BO	F	SCN1A	43	47	EMILIA ROMAGNA
13	Bellaria-BO	F	SCN1A	38	41	LOMBARDIA
14	Bellaria-BO	F	STXBP1	13	17	VENETO
15	Bellaria-BO	F	SCN8A	8	11	EMILIA ROMAGNA
16	Bellaria-BO	F	GRIN2A	9	11	CAMPANIA
17	Bellaria-BO	F	GABRG2	13	16	EMILIA ROMAGNA
18	Bellaria-BO	M	PCDH19	3	6	EMILIA ROMAGNA
19	Bellaria-BO	M	KCNQ2	5	7	EMILIA ROMAGNA
20	Bellaria-BO	M	SLC2A1	72	73	CAMPANIA
21	Bellaria-BO	M	SCN2A	17	19	CAMPANIA
22	Bellaria-BO	M	SCN2A	18	19	CAMPANIA
23	Bellaria-BO	F	SCN1A	11	11	EMILIA ROMAGNA
24	Bellaria-BO	F	SCN1A	29	31	LOMBARDIA
25	Bellaria-BO	M	CDKL5	1	2	.
26	Bellaria-BO	F	SCN1A	1	2	EMILIA ROMAGNA
27	Bellaria-BO	F	SLC6A1	54	55	EMILIA ROMAGNA
28	Bellaria-BO	M	MEF2C	46	46	MARCHE
29	Bellaria-BO	M	CHD2	28	28	MARCHE
30	Bellaria-BO	M	SLC2A1	21	30	PUGLIE
31	Bellaria-BO	F	MECP2	40	45	PUGLIE
32	Bellaria-BO	F	KCNQ2	21	22	EMILIA ROMAGNA
33	Bellaria-BO	F	KCNQ2	37	39	EMILIA ROMAGNA
34	Bellaria-BO	M	KCNQ2	23	24	EMILIA ROMAGNA
35	Bellaria-BO	M	SLC6A1	34	36	EMILIA ROMAGNA
36	Bellaria-BO	M	STXBP1	35	38	VENETO
37	Bellaria-BO	F	NEXMIF	26	28	LIGURIA
38	Bellaria-BO	F	NEXMIF	50	52	.
39	Bellaria-BO	F	NEXMIF	55	55	EMILIA ROMAGNA
40	Bellaria-BO	F	SMC1A	40	42	CALABRIA
41	Bellaria-BO	M	CACNA1E	26	29	TRENTINO A. A.
42	Bellaria-BO	F	KCNB1	53	55	EMILIA ROMAGNA
43	Bellaria-BO	M	SCN1A	59	61	TOSCANA
44	Bellaria-BO	M	STXBP1	34	36	PUGLIE
45	Bellaria-BO	F	GABRB2	47	49	EMILIA ROMAGNA
46	Bellaria-BO	F	YWHAG	66	68	CALABRIA
47	Bellaria-BO	M	GNAO1	12	14	EMILIA ROMAGNA
48	Bellaria-BO	M	SCN1A	25	25	CAMPANIA
49	Bellaria-BO	M	KCNT1	17	19	CAMPANIA
50	Bellaria-BO	M	SLC2A1	16	26	MARCHE
51	Bellaria-BO	F	SLC2A1	33	34	PUGLIE

52	Bellaria-BO	M	SCN1A	47	55	EMILIA ROMAGNA
53	Bellaria-BO	M	SCN1A	33	42	EMILIA ROMAGNA
54	Bellaria-BO	F	SCN1A	19	26	SICILIA
55	Bellaria-BO	M	SCN1A	14	21	PIEMONTE
56	Bellaria-BO	M	SCN1A	25	26	EMILIA ROMAGNA
57	Bellaria-BO	F	CYFIP2	32	32	LOMBARDIA
58	Bellaria-BO	F	MBD5	18	20	BASILICATA
59	Bellaria-BO	F	PARS2	24	25	UMBRIA
60	Bellaria-BO	F	PNKP	32	35	EMILIA ROMAGNA
61	Besta-MI	M	ARX	7	9	LOMBARDIA
62	Besta-MI	M	BRAT1	10	11	LOMBARDIA
63	Besta-MI	F	CACNA1A	4	8	LOMBARDIA
64	Besta-MI	M	CASK	6	9	LOMBARDIA
65	Besta-MI	M	CHD2	6	8	LOMBARDIA
66	Besta-MI	F	DNM1	9	13	LOMBARDIA
67	Besta-MI	F	FOXG1	8	12	PIEMONTE
68	Besta-MI	F	FOXG1	31	36	LOMBARDIA
69	Besta-MI	F	GABRA1	12	15	LOMBARDIA
70	Besta-MI	F	GABRA1	2	5	LOMBARDIA
71	Besta-MI	F	GABRA1	11	13	LOMBARDIA
72	Besta-MI	F	GABRB3	9	9	LOMBARDIA
73	Besta-MI	F	GABRG2	5	7	.
74	Besta-MI	F	GABRG2	18	20	LOMBARDIA
75	Besta-MI	M	GABRG2	6	6	LOMBARDIA
76	Besta-MI	F	GNAO1	6	8	LOMBARDIA
77	Besta-MI	F	GRIN2A	6	8	LOMBARDIA
78	Besta-MI	M	GRIN2A	23	28	LOMBARDIA
79	Besta-MI	M	GRIN2A	5	8	LOMBARDIA
80	Besta-MI	F	GRIN2A	16	20	.
81	Besta-MI	F	KCNA2	10	13	LOMBARDIA
82	Besta-MI	M	KCNA2	23	27	PIEMONTE
83	Besta-MI	M	KCNA2	15	17	PIEMONTE
84	Besta-MI	M	KCNA2	35	36	LOMBARDIA
85	Besta-MI	F	KCNQ2	8	12	PIEMONTE
86	Besta-MI	F	KCNQ2	0	1	LOMBARDIA
87	Besta-MI	F	KCNQ2	25	29	PIEMONTE
88	Besta-MI	F	KCNT1	53	56	LOMBARDIA
89	Besta-MI	F	KCNT1	17	19	PUGLIE
90	Besta-MI	M	KCNT1	0	6	EMILIA ROMAGNA
91	Besta-MI	M	KCNT1	5	6	LOMBARDIA
92	Besta-MI	F	MECP2	3	5	LOMBARDIA
93	Besta-MI	M	MECP2	17	22	LOMBARDIA
94	Besta-MI	M	MEF2C	5	10	LOMBARDIA
95	Besta-MI	F	PCDH19	5	8	LOMBARDIA
96	Besta-MI	F	PCDH19	16	18	LOMBARDIA
97	Besta-MI	F	SCN1A	3	9	LOMBARDIA
98	Besta-MI	F	SCN1A	2	7	.
99	Besta-MI	F	SCN1A	38	42	CALABRIA
100	Besta-MI	F	SCN1A	4	6	LOMBARDIA
101	Besta-MI	M	SCN1A	15	21	LOMBARDIA
102	Besta-MI	F	SCN1A	2	3	CALABRIA
103	Besta-MI	F	SCN1A	0	0	LOMBARDIA

104	Besta-MI	M	SCN1A	15	16	PUGLIE
105	Besta-MI	M	SCN1A	9	11	ABRUZZI
106	Besta-MI	F	SCN1A	6	6	LOMBARDIA
107	Besta-MI	F	SCN2A	9	11	CAMPANIA
108	Besta-MI	M	SCN2A	7	13	LOMBARDIA
109	Besta-MI	M	SCN8A	17	20	VALLE DÕAOSTA
110	Besta-MI	M	SCN8A	1	4	LOMBARDIA
111	Besta-MI	M	SCN8A	5	9	LOMBARDIA
112	Besta-MI	F	SCN8A	23	28	.
113	Besta-MI	M	SCN8A	3	6	LOMBARDIA
114	Besta-MI	M	SLC12A5	4	8	LOMBARDIA
115	Besta-MI	M	SLC2A1	27	30	LOMBARDIA
116	Besta-MI	F	SLC2A1	10	16	SICILIA
117	Besta-MI	M	SLC6A1	17	19	LOMBARDIA
118	Besta-MI	M	SLC6A1	9	9	LOMBARDIA
119	Besta-MI	M	SYNGAP1	9	12	LOMBARDIA
120	Besta-MI	M	SYNGAP1	11	16	LOMBARDIA
121	Besta-MI	F	SYNGAP1	13	16	LOMBARDIA
122	Besta-MI	F	TBC1D24	5	11	LOMBARDIA
123	Besta-MI	F	TBC1D24	28	34	LOMBARDIA
124	Besta-MI	M	CDKL5	4	12	CALABRIA
125	Besta-MI	M	KCNB1	10	14	VENETO
126	Besta-MI	M	ARHGEF9	15	18	LOMBARDIA
127	Besta-MI	F	GABRA5	8	9	LOMBARDIA
128	Besta-MI	M	HNRNPU	8	8	LAZIO
129	Besta-MI	M	SCN1B	8	10	LOMBARDIA
130	Besta-MI	M	SCN3A	2	2	LOMBARDIA
131	Besta-MI	F	SLC13A5	16	19	SICILIA
132	Besta-MI	F	SLC13A5	17	20	.
133	Besta-MI	M	SLC13A5	5	9	PUGLIE
134	Besta-MI	F	ST3GAL5	8	12	PUGLIE
135	Gaslini-GE	M	STXBP1	4	11	LOMBARDIA
136	Gaslini-GE	F	STXBP1	4	11	CAMPANIA
137	Gaslini-GE	M	KCNT1	15	22	LIGURIA
138	Gaslini-GE	F	CDKL5	2	9	LIGURIA
139	Gaslini-GE	F	SCN1A	6	13	LIGURIA
140	Gaslini-GE	M	STXBP1	5	12	EMILIA ROMAGNA
141	Gaslini-GE	F	KCNQ2	2	9	LOMBARDIA
142	Gaslini-GE	M	TBC1D24	11	18	LAZIO
143	Gaslini-GE	F	TBC1D24	2	9	SICILIA
144	Gaslini-GE	F	SCN1A	1	8	LIGURIA
145	Gaslini-GE	F	SCN8A	7	14	LIGURIA
146	Gaslini-GE	F	CDKL5	1	8	PIEMONTE
147	Gaslini-GE	F	SCN1A	1	8	CAMPANIA
148	Gaslini-GE	F	KCNQ2	0	7	LIGURIA
149	Gaslini-GE	M	CDKL5	12	19	LIGURIA
150	Gaslini-GE	M	SCN8A	2	9	CALABRIA
151	Gaslini-GE	F	SCN8A	5	11	.
152	Gaslini-GE	F	SLC2A1	5	11	LIGURIA
153	Gaslini-GE	M	KCNQ2	1	7	SICILIA
154	Gaslini-GE	F	CHD2	14	20	PIEMONTE
155	Gaslini-GE	F	CDKL5	0	6	LIGURIA

156	Gaslini-GE	F	CDKL5	2	8	PIEMONTE
157	Gaslini-GE	M	KCNT1	3	9	LIGURIA
158	Gaslini-GE	M	KCNQ2	0	6	LIGURIA
159	Gaslini-GE	M	STXBP1	6	12	PIEMONTE
160	Gaslini-GE	M	KCNQ2	1	7	LIGURIA
161	Gaslini-GE	F	SCN1A	8	14	SICILIA
162	Gaslini-GE	M	KCNQ2	6	12	UMBRIA
163	Gaslini-GE	F	SLC25A22	0	6	SICILIA
164	Gaslini-GE	F	KCNQ2	22	27	VENETO
165	Gaslini-GE	M	HCN1	2	7	LIGURIA
166	Gaslini-GE	F	PCDH19	11	16	EMILIA ROMAGNA
167	Gaslini-GE	M	SLC6A1	9	14	EMILIA ROMAGNA
168	Gaslini-GE	M	GRIN2A	13	18	SICILIA
169	Gaslini-GE	F	PCDH19	1	6	LIGURIA
170	Gaslini-GE	M	SLC2A1	9	13	PIEMONTE
171	Gaslini-GE	F	PACS2	0	4	CAMPANIA
172	Gaslini-GE	M	EEF1A2	1	5	LIGURIA
173	Gaslini-GE	M	NTRK2	1	5	.
174	Gaslini-GE	M	KCNB1	0	4	LIGURIA
175	Gaslini-GE	M	SZT2	6	10	SICILIA
176	Gaslini-GE	M	KCNQ2	0	3	CALABRIA
177	Gaslini-GE	F	GABRB3	1	4	SICILIA
178	Gaslini-GE	F	GRIN2A	10	13	LIGURIA
179	Gaslini-GE	M	CDKL5	0	3	SARDEGNA
180	Gaslini-GE	M	GABRA1	9	12	SICILIA
181	Gaslini-GE	M	SYNGAP1	4	6	EMILIA ROMAGNA
182	Gaslini-GE	F	GABRG2	14	16	LIGURIA
183	Gaslini-GE	M	SPTAN1	15	17	PIEMONTE
184	Gaslini-GE	M	GRIN2A	7	9	PUGLIE
185	Gaslini-GE	M	SCN1A	4	5	.
186	Gaslini-GE	M	SCN2A	1	2	CAMPANIA
187	Gaslini-GE	F	SYNGAP1	5	5	PIEMONTE
188	Gaslini-GE	M	GRIN2A	9	9	LIGURIA
189	Gaslini-GE	F	SCN2A	15	15	LIGURIA
190	Gaslini-GE	F	GRIN2A	13	13	LIGURIA
191	Gaslini-GE	F	SCN1A	12	17	EMILIA ROMAGNA
192	Gaslini-GE	M	SCN1A	2	8	TOSCANA
193	Gaslini-GE	M	GRIN1	1	5	PUGLIE
194	Gaslini-GE	F	CHD2	6	6	LIGURIA
195	Gaslini-GE	M	KCNT1	0	3	LIGURIA
196	Gemelli-RM	F	SYNGAP1	17	20	LAZIO
197	Gemelli-RM	M	SYNGAP1	16	18	LAZIO
198	Gemelli-RM	F	ARX	15	17	PUGLIE
199	Gemelli-RM	F	SLC6A1	13	16	ABRUZZI
200	Gemelli-RM	F	SCN1A	12	16	SARDEGNA
201	Gemelli-RM	F	SCN1A	11	16	LAZIO
202	Gemelli-RM	M	SPTAN1	20	26	LAZIO
203	Gemelli-RM	M	SPTAN1	9	12	CAMPANIA
204	Gemelli-RM	F	SLC2A1	6	15	LAZIO
205	Gemelli-RM	F	SLC6A1	8	13	CAMPANIA
206	Gemelli-RM	F	KCNQ2	5	10	ABRUZZI
207	Gemelli-RM	M	DOCK7	11	17	CALABRIA

208	Gemelli-RM	F	CACNA1E	3	7	ABRUZZI
209	Gemelli-RM	F	SYNGAP1	12	15	LAZIO
210	Gemelli-RM	M	CASK	10	16	LAZIO
211	Gemelli-RM	F	TBC1D24	17	23	LAZIO
212	Gemelli-RM	F	SCN2A	17	20	LAZIO
213	Gemelli-RM	F	MECP2	2	5	LAZIO
214	Gemelli-RM	F	PCDH19	15	25	LAZIO
215	Gemelli-RM	M	SYNGAP1	10	13	LAZIO
216	Gemelli-RM	M	GABRB3	4	8	LAZIO
217	Gemelli-RM	F	SLC2A1	6	11	LAZIO
218	Gemelli-RM	M	DNM1L	8	13	LAZIO
219	Gemelli-RM	F	PCDH19	1	10	LAZIO
220	Gemelli-RM	F	TBC1D24	4	7	LAZIO
221	Gemelli-RM	F	MEF2C	7	17	LAZIO
222	Gemelli-RM	M	SCN1A	12	15	ABRUZZI
223	Gemelli-RM	F	SCN1A	2	11	ABRUZZI
224	Gemelli-RM	M	SCN1A	11	18	.
225	Gemelli-RM	M	SCN1A	6	14	LAZIO
226	Gemelli-RM	M	SCN1A	1	5	LAZIO
227	Gemelli-RM	F	SCN8A	1	2	LAZIO
228	Gemelli-RM	F	SCN1A	6	14	.
229	Gemelli-RM	F	SCN1A	4	14	.
230	Gemelli-RM	M	SCN1A	2	12	LAZIO
231	Gemelli-RM	F	SCN1A	4	6	LAZIO
232	Gemelli-RM	F	PCDH19	11	13	LAZIO
233	Gemelli-RM	F	SCN1A	8	13	ABRUZZI
234	Gemelli-RM	F	GABRA1	11	12	LAZIO
235	Gemelli-RM	F	GABRA1	9	12	LAZIO
236	Gemelli-RM	M	STXBP1	5	12	LAZIO
237	Gemelli-RM	M	GABRA1	8	11	CALABRIA
238	Gemelli-RM	F	SCN1A	8	10	LAZIO
239	Gemelli-RM	M	KCNQ2	5	10	CALABRIA
240	Gemelli-RM	F	CDKL5	1	10	SICILIA
241	Gemelli-RM	F	SCN1A	2	9	CAMPANIA
242	Gemelli-RM	F	CDKL5	1	9	LAZIO
243	Gemelli-RM	F	GNAO1	2	8	LAZIO
244	Gemelli-RM	F	SCN1A	1	8	UMBRIA
245	Gemelli-RM	M	SCN1A	4	8	.
246	Gemelli-RM	M	KCNQ2	1	6	LAZIO
247	Gemelli-RM	M	KCNT1	1	6	LAZIO
248	Gemelli-RM	F	SCN1A	1	4	PUGLIE
249	Gemelli-RM	F	FOXG1	0	3	LAZIO
250	Gemelli-RM	F	SCN2A	1	2	LAZIO
251	Gemelli-RM	F	SCN8A	0	2	LAZIO
252	Gemelli-RM	F	CDKL5	2	2	CAMPANIA
253	Gemelli-RM	F	CDKL5	20	27	CALABRIA
254	Gemelli-RM	M	GRIN1	21	25	LAZIO
255	Gemelli-RM	F	KCNQ2	17	24	LAZIO
256	IRCCSMEDEALC	F	ATP1A2	10	10	LOMBARDIA
257	IRCCSMEDEALC	F	ATP1A3	12	13	.
258	IRCCSMEDEALC	M	ATP1A3	2	9	LOMBARDIA
259	IRCCSMEDEALC	M	BRAT1	42	44	LOMBARDIA

260	IRCCSMEDEALC	F	CACNA1A	5	8	PUGLIE
261	IRCCSMEDEALC	M	CACNA1A	9	10	LOMBARDIA
262	IRCCSMEDEALC	M	CACNA1A	17	21	LOMBARDIA
263	IRCCSMEDEALC	M	CASK	5	8	LOMBARDIA
264	IRCCSMEDEALC	F	CHD2	5	8	VENETO
265	IRCCSMEDEALC	M	CHD2	30	31	LOMBARDIA
266	IRCCSMEDEALC	M	CHD2	12	16	LOMBARDIA
267	IRCCSMEDEALC	M	CHD2	3	7	LOMBARDIA
268	IRCCSMEDEALC	M	CLCN4	18	23	LOMBARDIA
269	IRCCSMEDEALC	F	DNM1L	20	22	LOMBARDIA
270	IRCCSMEDEALC	M	EEF1A2	13	17	PUGLIE
271	IRCCSMEDEALC	F	FOXG1	1	10	LOMBARDIA
272	IRCCSMEDEALC	F	FOXG1	5	10	PUGLIE
273	IRCCSMEDEALC	F	FOXG1	1	9	TOSCANA
274	IRCCSMEDEALC	F	GABRA1	25	30	CAMPANIA
275	IRCCSMEDEALC	F	GABRA1	17	18	PUGLIE
276	IRCCSMEDEALC	F	GABRA1	12	18	PUGLIE
277	IRCCSMEDEALC	F	GABRB3	33	34	LOMBARDIA
278	IRCCSMEDEALC	F	GABRG2	2	6	VENETO
279	IRCCSMEDEALC	M	GRIN1	10	16	LOMBARDIA
280	IRCCSMEDEALC	M	GRIN2A	11	16	LOMBARDIA
281	IRCCSMEDEALC	M	GRIN2A	10	16	PUGLIE
282	IRCCSMEDEALC	M	GRIN2A	10	16	LOMBARDIA
283	IRCCSMEDEALC	M	GRIN2B	44	46	VENETO
284	IRCCSMEDEALC	M	GRIN2B	1	7	LOMBARDIA
285	IRCCSMEDEALC	F	KCNB1	6	6	.
286	IRCCSMEDEALC	M	KCNB1	14	18	.
287	IRCCSMEDEALC	F	KCNQ2	24	25	PIEMONTE
288	IRCCSMEDEALC	F	KCNQ2	0	2	LOMBARDIA
289	IRCCSMEDEALC	M	KCNQ2	1	1	LOMBARDIA
290	IRCCSMEDEALC	F	KCNQ2	19	21	BASILICATA
291	IRCCSMEDEALC	F	KCNQ2	1	5	LOMBARDIA
292	IRCCSMEDEALC	M	KCNT1	8	15	PUGLIE
293	IRCCSMEDEALC	F	MECP2	2	5	LOMBARDIA
294	IRCCSMEDEALC	F	MECP2	1	2	PUGLIE
295	IRCCSMEDEALC	F	MECP2	3	5	PUGLIE
296	IRCCSMEDEALC	F	MECP2	2	2	LOMBARDIA
297	IRCCSMEDEALC	F	MECP2	9	10	CALABRIA
298	IRCCSMEDEALC	M	NEXMIF	11	13	LOMBARDIA
299	IRCCSMEDEALC	F	NEXMIF	15	20	PUGLIE
300	IRCCSMEDEALC	F	NEXMIF	21	27	LOMBARDIA
301	IRCCSMEDEALC	M	NEXMIF	19	19	LOMBARDIA
302	IRCCSMEDEALC	F	PCDH19	17	17	LOMBARDIA
303	IRCCSMEDEALC	M	SCN1A	1	3	LOMBARDIA
304	IRCCSMEDEALC	F	SCN1A	0	2	LOMBARDIA
305	IRCCSMEDEALC	F	SCN1A	1	5	VENETO
306	IRCCSMEDEALC	F	SCN1A	1	7	LOMBARDIA
307	IRCCSMEDEALC	F	SCN1A	2	7	LOMBARDIA
308	IRCCSMEDEALC	F	SCN1A	1	4	LOMBARDIA
309	IRCCSMEDEALC	F	SCN1A	3	3	PUGLIE
310	IRCCSMEDEALC	M	SCN1A	48	52	LOMBARDIA
311	IRCCSMEDEALC	M	SCN1A	2	6	LOMBARDIA

312	IRCCSMEDEALC	F	SCN1A	1	2	LOMBARDIA
313	IRCCSMEDEALC	F	SCN1A	6	6	LOMBARDIA
314	IRCCSMEDEALC	M	SCN2A	17	18	LOMBARDIA
315	IRCCSMEDEALC	M	SCN2A	26	29	SICILIA
316	IRCCSMEDEALC	M	SCN2A	5	9	SICILIA
317	IRCCSMEDEALC	F	SCN8A	12	19	LOMBARDIA
318	IRCCSMEDEALC	M	SCN8A	8	14	LOMBARDIA
319	IRCCSMEDEALC	F	SLC2A1	2	3	LOMBARDIA
320	IRCCSMEDEALC	M	SLC6A1	6	10	LOMBARDIA
321	IRCCSMEDEALC	M	SLC6A1	14	22	LOMBARDIA
322	IRCCSMEDEALC	M	SLC6A1	7	10	VENETO
323	IRCCSMEDEALC	M	SLC9A6	14	20	SICILIA
324	IRCCSMEDEALC	M	SLC9A6	11	16	CALABRIA
325	IRCCSMEDEALC	M	SPTAN1	6	9	LOMBARDIA
326	IRCCSMEDEALC	M	SPTAN1	37	39	VENETO
327	IRCCSMEDEALC	M	STXBP1	35	37	VENETO
328	IRCCSMEDEALC	F	STXBP1	13	17	VENETO
329	IRCCSMEDEALC	M	STXBP1	10	15	PUGLIE
330	IRCCSMEDEALC	F	SYNGAP1	7	12	.
331	IRCCSMEDEALC	F	UBA5	16	18	FRIULI V. G.
332	IRCCSMEDEALC	F	UBA5	22	27	PUGLIE
333	IRCCSMEDEALC	M	UBA5	45	47	LOMBARDIA
334	IRCCSMEDEALC	F	YWHAG	3	3	PUGLIE
335	IRCCSMEDEALC	M	GRIN1	10	16	LOMBARDIA
336	IRCCSMEDEALC	M	STXBP1	35	37	VENETO
337	IRCCSMEDEALC	M	CLTC	24	28	LOMBARDIA
338	IRCCSMEDEALC	M	POLG	26	26	FRIULI V. G.
339	IRCCSMEDEALC	F	SCN3A	13	18	LOMBARDIA
340	IRCCSMEDEALC	M	HNRNPU	13	17	LOMBARDIA
341	IRCCSMEDEALC	F	PURA	24	24	VENETO
342	IRCCSMEDEALC	M	MBD5	14	19	LOMBARDIA
343	IRCCSMEDEALC	M	PURA	15	21	LOMBARDIA
344	IRCCSMEDEALC	F	PNPO	21	21	MARCHE
345	IRCCSMEDEALC	F	CSNK2B	8	11	LOMBARDIA
346	IRCCSMEDEALC	M	STXBP1	12	12	ABRUZZI
347	MaterDomini-CZ	M	SCN1A	9	13	CALABRIA
348	MaterDomini-CZ	F	CHD2	63	64	CALABRIA
349	MaterDomini-CZ	M	KCNT1	34	35	CALABRIA
350	MaterDomini-CZ	F	PCDH19	52	53	CALABRIA
351	MaterDomini-CZ	F	CHD2	20	21	LOMBARDIA
352	MaterDomini-CZ	F	KCNQ2	31	32	CALABRIA
353	MaterDomini-CZ	F	SCN1A	74	75	CALABRIA
354	MaterDomini-CZ	M	KCNA2	36	38	CAMPANIA
355	MaterDomini-CZ	F	KCNT1	36	38	CALABRIA
356	MaterDomini-CZ	F	KCNQ2	18	20	CALABRIA
357	MaterDomini-CZ	F	SCN1A	18	21	CALABRIA
358	MaterDomini-CZ	M	SCN1A	13	16	CALABRIA
359	MaterDomini-CZ	F	SCN1A	13	16	CALABRIA
360	MaterDomini-CZ	M	SCN1A	16	19	CALABRIA
361	MaterDomini-CZ	M	SCN1A	22	26	CALABRIA
362	MaterDomini-CZ	F	SLC2A1	8	13	CALABRIA
363	Meyer-FI	F	SLC6A1	20	22	VENETO

364	Meyer-FI	F	SPTAN1	13	16	LAZIO
365	Meyer-FI	F	ST3GAL5	13	16	PUGLIE
366	Meyer-FI	M	GRIN1	13	16	PIEMONTE
367	Meyer-FI	F	GRIN1	13	15	CALABRIA
368	Meyer-FI	M	CDKL5	8	14	PUGLIE
369	Meyer-FI	M	GABRA5	14	14	MOLISE
370	Meyer-FI	M	SZT2	7	13	ABRUZZI
371	Meyer-FI	F	SYNGAP1	8	12	MARCHE
372	Meyer-FI	F	ALG13	8	9	UMBRIA
373	Meyer-FI	F	ALG13	3	6	EMILIA ROMAGNA
374	Meyer-FI	M	ARX	10	18	.
375	Meyer-FI	M	ARX	2	10	CALABRIA
376	Meyer-FI	M	ARX	3	8	EMILIA ROMAGNA
377	Meyer-FI	M	ATP1A2	3	10	LOMBARDIA
378	Meyer-FI	F	ATP1A3	3	9	ABRUZZI
379	Meyer-FI	F	ATP1A3	7	9	SICILIA
380	Meyer-FI	F	ATP1A3	18	20	CAMPANIA
381	Meyer-FI	M	ATP1A3	26	30	EMILIA ROMAGNA
382	Meyer-FI	M	ATP1A3	7	9	.
383	Meyer-FI	F	ATP1A3	1	3	LOMBARDIA
384	Meyer-FI	F	ATP1A3	3	10	EMILIA ROMAGNA
385	Meyer-FI	F	ATP6V1A	5	8	PUGLIE
386	Meyer-FI	F	ATP6V1A	15	19	TOSCANA
387	Meyer-FI	M	BRAT1	0	1	TOSCANA
388	Meyer-FI	F	CACNA1A	10	15	MARCHE
389	Meyer-FI	F	CACNA1A	2	7	CAMPANIA
390	Meyer-FI	F	CACNA1A	6	11	TOSCANA
391	Meyer-FI	F	CACNA1A	2	6	TOSCANA
392	Meyer-FI	F	CACNA1A	15	18	LAZIO
393	Meyer-FI	M	CACNA1A	19	22	.
394	Meyer-FI	F	CACNA1A	17	19	LAZIO
395	Meyer-FI	F	CACNA1A	23	25	TOSCANA
396	Meyer-FI	M	CACNA1A	11	13	TOSCANA
397	Meyer-FI	M	CACNA1A	2	2	PIEMONTE
398	Meyer-FI	M	CACNA1A	9	9	TRENTINO A. A.
399	Meyer-FI	M	CACNA1A	9	9	EMILIA ROMAGNA
400	Meyer-FI	F	CACNA1E	12	15	BASILICATA
401	Meyer-FI	F	CACNA1E	20	23	TOSCANA
402	Meyer-FI	F	CACNA1E	3	5	.
403	Meyer-FI	F	CASK	14	20	TOSCANA
404	Meyer-FI	F	CASK	2	8	CAMPANIA
405	Meyer-FI	F	CASK	2	7	TOSCANA
406	Meyer-FI	F	CASK	9	14	.
407	Meyer-FI	F	CASK	16	21	LOMBARDIA
408	Meyer-FI	F	CASK	2	6	TOSCANA
409	Meyer-FI	F	CDKL5	1	9	SICILIA
410	Meyer-FI	M	CDKL5	7	14	VENETO
411	Meyer-FI	F	CDKL5	7	14	MARCHE
412	Meyer-FI	F	CDKL5	1	7	TRENTINO A. A.
413	Meyer-FI	F	NEXMIF	5	6	PUGLIE
414	Meyer-FI	M	CDKL5	4	13	TOSCANA
415	Meyer-FI	F	CDKL5	2	9	VALLE DÕAOSTA

416	Meyer-FI	F	CDKL5	11	17	LAZIO
417	Meyer-FI	F	CDKL5	0	5	TOSCANA
418	Meyer-FI	F	CDKL5	1	7	TOSCANA
419	Meyer-FI	F	CDKL5	10	13	CALABRIA
420	Meyer-FI	F	CDKL5	3	6	PIEMONTE
421	Meyer-FI	F	CDKL5	4	7	CAMPANIA
422	Meyer-FI	F	CDKL5	1	3	CAMPANIA
423	Meyer-FI	F	CDKL5	0	0	TOSCANA
424	Meyer-FI	F	CDKL5	0	0	PIEMONTE
425	Meyer-FI	F	CDKL5	1	1	CAMPANIA
426	Meyer-FI	F	CDKL5	2	2	VENETO
427	Meyer-FI	F	CDKL5	2	2	TOSCANA
428	Meyer-FI	F	CDKL5	2	3	UMBRIA
429	Meyer-FI	F	CDKL5	14	15	EMILIA ROMAGNA
430	Meyer-FI	M	CHD2	18	24	TOSCANA
431	Meyer-FI	M	CHD2	15	19	.
432	Meyer-FI	F	CHD2	4	8	CALABRIA
433	Meyer-FI	M	CHD2	11	14	EMILIA ROMAGNA
434	Meyer-FI	F	CHD2	10	13	CAMPANIA
435	Meyer-FI	M	CHD2	29	31	CAMPANIA
436	Meyer-FI	M	CHD2	14	16	.
437	Meyer-FI	M	CHD2	20	25	TOSCANA
438	Meyer-FI	M	CHD2	7	9	.
439	Meyer-FI	M	CHD2	7	10	.
440	Meyer-FI	F	CHD2	5	5	UMBRIA
441	Meyer-FI	F	CHD2	7	7	SICILIA
442	Meyer-FI	F	CLCN4	3	6	TOSCANA
443	Meyer-FI	F	DNM1	42	43	TOSCANA
444	Meyer-FI	M	DNM1	36	36	UMBRIA
445	Meyer-FI	F	EEF1A2	4	6	SICILIA
446	Meyer-FI	M	EEF1A2	6	8	TOSCANA
447	Meyer-FI	F	EEF1A2	0	2	TRENTINO A. A.
448	Meyer-FI	M	EEF1A2	2	3	EMILIA ROMAGNA
449	Meyer-FI	F	EEF1A2	2	6	VENETO
450	Meyer-FI	M	EEF1A2	6	6	TOSCANA
451	Meyer-FI	F	FGF12	12	14	TOSCANA
452	Meyer-FI	M	FOXG1	8	13	CAMPANIA
453	Meyer-FI	F	FOXG1	11	14	SICILIA
454	Meyer-FI	M	FOXG1	22	25	LOMBARDIA
455	Meyer-FI	F	FOXG1	9	11	CAMPANIA
456	Meyer-FI	M	FOXG1	2	4	LAZIO
457	Meyer-FI	F	FOXG1	2	3	SICILIA
458	Meyer-FI	F	FOXG1	2	2	SICILIA
459	Meyer-FI	M	FOXG1	9	19	LOMBARDIA
460	Meyer-FI	F	FOXG1	6	13	PUGLIE
461	Meyer-FI	M	FOXG1	3	11	TOSCANA
462	Meyer-FI	F	GABRA1	12	17	CAMPANIA
463	Meyer-FI	F	GABRA1	22	25	.
464	Meyer-FI	F	GABRA1	11	14	CALABRIA
465	Meyer-FI	F	GABRA1	14	16	.
466	Meyer-FI	F	GABRA1	9	10	SICILIA
467	Meyer-FI	M	GABRA1	15	16	PIEMONTE

468	Meyer-FI	F	GABRA1	1	1	PIEMONTE
469	Meyer-FI	M	GABRA1	1	1	LAZIO
470	Meyer-FI	M	GABRA1	1	1	LOMBARDIA
471	Meyer-FI	F	GABRB1	5	9	ABRUZZI
472	Meyer-FI	F	GABRB2	4	8	PIEMONTE
473	Meyer-FI	F	GABRB2	12	14	PUGLIE
474	Meyer-FI	F	GABRB2	16	16	LOMBARDIA
475	Meyer-FI	F	GABRB3	0	7	VENETO
476	Meyer-FI	M	GABRB3	1	4	TOSCANA
477	Meyer-FI	M	GABRB3	15	18	MARCHE
478	Meyer-FI	M	GABRB3	2	5	PUGLIE
479	Meyer-FI	F	GABRB3	3	3	LAZIO
480	Meyer-FI	F	GABRB3	0	0	VENETO
481	Meyer-FI	M	GABRB3	16	16	VENETO
482	Meyer-FI	F	GABRB3	1	1	UMBRIA
483	Meyer-FI	F	GABRG2	7	11	TOSCANA
484	Meyer-FI	M	GABRG2	30	33	VENETO
485	Meyer-FI	F	GABRG2	1	3	EMILIA ROMAGNA
486	Meyer-FI	F	GABRG2	2	4	TOSCANA
487	Meyer-FI	M	GABRG2	8	10	PUGLIE
488	Meyer-FI	F	GABRG2	7	8	EMILIA ROMAGNA
489	Meyer-FI	F	GABRG2	12	15	SARDEGNA
490	Meyer-FI	M	GABRG2	5	12	TOSCANA
491	Meyer-FI	F	GABRG2	9	13	VENETO
492	Meyer-FI	F	GABRG2	9	11	SICILIA
493	Meyer-FI	M	GABRG2	14	16	LAZIO
494	Meyer-FI	F	GABRG2	4	5	LAZIO
495	Meyer-FI	F	GABRG2	12	12	MARCHE
496	Meyer-FI	F	GABRG2	6	6	PIEMONTE
497	Meyer-FI	M	GABRG2	5	5	LOMBARDIA
498	Meyer-FI	F	GABRG2	9	9	LOMBARDIA
499	Meyer-FI	F	GABRG2	9	9	TOSCANA
500	Meyer-FI	F	GABRG2	2	2	UMBRIA
501	Meyer-FI	F	GNAO1	16	22	PIEMONTE
502	Meyer-FI	F	GNAO1	12	17	PUGLIE
503	Meyer-FI	M	GNAO1	14	17	TOSCANA
504	Meyer-FI	M	GNAO1	3	4	ABRUZZI
505	Meyer-FI	F	GNAO1	13	14	TOSCANA
506	Meyer-FI	F	GNAO1	3	3	SICILIA
507	Meyer-FI	F	GNAO1	14	14	LOMBARDIA
508	Meyer-FI	M	GNAO1	13	13	EMILIA ROMAGNA
509	Meyer-FI	F	GNAO1	2	2	TOSCANA
510	Meyer-FI	M	STXBP1	1	9	ABRUZZI
511	Meyer-FI	F	GRIN1	6	10	SICILIA
512	Meyer-FI	M	GRIN2A	22	29	TOSCANA
513	Meyer-FI	F	GRIN2A	16	22	TOSCANA
514	Meyer-FI	M	GRIN2A	9	15	PUGLIE
515	Meyer-FI	M	GRIN2A	7	12	UMBRIA
516	Meyer-FI	M	GRIN2A	8	11	LAZIO
517	Meyer-FI	F	GRIN2A	16	18	CALABRIA
518	Meyer-FI	M	GRIN2A	10	12	CAMPANIA
519	Meyer-FI	M	GRIN2A	21	24	LOMBARDIA

520	Meyer-FI	M	GRIN2A	14	18	TRENTINO A. A.
521	Meyer-FI	F	GRIN2A	9	13	TOSCANA
522	Meyer-FI	F	GRIN2A	12	18	SICILIA
523	Meyer-FI	M	GRIN2A	9	10	TOSCANA
524	Meyer-FI	M	GRIN2A	8	8	TOSCANA
525	Meyer-FI	F	GRIN2A	12	13	.
526	Meyer-FI	M	GRIN2A	6	7	TOSCANA
527	Meyer-FI	F	GRIN2A	8	8	TOSCANA
528	Meyer-FI	M	GRIN2A	43	43	LAZIO
529	Meyer-FI	M	GRIN2B	4	8	LAZIO
530	Meyer-FI	M	GRIN2B	1	4	CAMPANIA
531	Meyer-FI	M	GRIN2B	1	5	PIEMONTE
532	Meyer-FI	F	GRIN2B	7	10	EMILIA ROMAGNA
533	Meyer-FI	F	GRIN2B	2	4	CAMPANIA
534	Meyer-FI	M	GRIN2B	6	12	TOSCANA
535	Meyer-FI	M	GRIN2B	8	12	TOSCANA
536	Meyer-FI	M	GRIN2B	13	13	VENETO
537	Meyer-FI	F	HCN1	22	26	UMBRIA
538	Meyer-FI	M	HCN1	14	20	SICILIA
539	Meyer-FI	F	HCN1	5	11	TOSCANA
540	Meyer-FI	F	GRIN1	4	8	SICILIA
541	Meyer-FI	M	HCN1	7	10	VENETO
542	Meyer-FI	F	KCNA2	3	10	TOSCANA
543	Meyer-FI	F	KCNA2	6	11	.
544	Meyer-FI	M	KCNA2	14	19	MARCHE
545	Meyer-FI	F	KCNA2	18	22	LAZIO
546	Meyer-FI	M	KCNA2	2	2	VENETO
547	Meyer-FI	F	KCNB1	11	17	TRENTINO A. A.
548	Meyer-FI	F	KCNB1	23	28	SICILIA
549	Meyer-FI	F	KCNB1	7	13	UMBRIA
550	Meyer-FI	M	KCNB1	6	12	LIGURIA
551	Meyer-FI	F	KCNB1	8	13	EMILIA ROMAGNA
552	Meyer-FI	M	KCNB1	17	22	PUGLIE
553	Meyer-FI	M	KCNB1	2	6	LOMBARDIA
554	Meyer-FI	M	KCNB1	2	6	CALABRIA
555	Meyer-FI	M	KCNB1	9	12	LOMBARDIA
556	Meyer-FI	F	KCNB1	4	4	MARCHE
557	Meyer-FI	F	KCNQ2	6	14	EMILIA ROMAGNA
558	Meyer-FI	F	KCNQ2	1	9	LOMBARDIA
559	Meyer-FI	M	KCNQ2	1	9	PIEMONTE
560	Meyer-FI	F	KCNQ2	8	16	CAMPANIA
561	Meyer-FI	F	KCNQ2	1	9	TOSCANA
562	Meyer-FI	F	KCNQ2	19	26	EMILIA ROMAGNA
563	Meyer-FI	F	KCNQ2	1	8	CAMPANIA
564	Meyer-FI	F	KCNQ2	3	8	LOMBARDIA
565	Meyer-FI	F	KCNQ2	9	15	EMILIA ROMAGNA
566	Meyer-FI	M	KCNQ2	0	4	EMILIA ROMAGNA
567	Meyer-FI	F	KCNQ2	3	7	LIGURIA
568	Meyer-FI	F	KCNQ2	2	11	PIEMONTE
569	Meyer-FI	M	KCNQ2	1	2	TOSCANA
570	Meyer-FI	F	KCNQ2	2	3	MARCHE
571	Meyer-FI	M	KCNQ2	1	2	ABRUZZI

572	Meyer-FI	M	KCNQ2	1	3	MARCHE
573	Meyer-FI	M	KCNQ2	14	16	CAMPANIA
574	Meyer-FI	M	KCNQ2	16	18	PUGLIE
575	Meyer-FI	F	KCNQ2	1	4	TOSCANA
576	Meyer-FI	M	KCNQ2	2	5	VENETO
577	Meyer-FI	F	KCNQ2	0	4	TOSCANA
578	Meyer-FI	M	KCNQ2	5	10	TOSCANA
579	Meyer-FI	M	KCNQ2	6	6	EMILIA ROMAGNA
580	Meyer-FI	M	KCNQ2	10	10	MARCHE
581	Meyer-FI	F	KCNQ2	12	13	.
582	Meyer-FI	F	KCNQ2	1	1	SICILIA
583	Meyer-FI	F	KCNQ2	1	1	TOSCANA
584	Meyer-FI	F	KCNQ2	1	2	PIEMONTE
585	Meyer-FI	F	KCNQ2	0	3	LOMBARDIA
586	Meyer-FI	F	KCNT1	1	8	CAMPANIA
587	Meyer-FI	M	KCNT1	5	11	LOMBARDIA
588	Meyer-FI	F	KCNT1	6	12	SARDEGNA
589	Meyer-FI	F	KCNT1	7	13	LAZIO
590	Meyer-FI	F	KCNT1	3	9	.
591	Meyer-FI	F	KCNT1	14	20	TOSCANA
592	Meyer-FI	M	KCNT1	27	28	TOSCANA
593	Meyer-FI	M	KCNT1	8	12	PUGLIE
594	Meyer-FI	M	KCNT1	0	4	.
595	Meyer-FI	M	KCNT1	1	4	CAMPANIA
596	Meyer-FI	F	KCNT1	38	41	LOMBARDIA
597	Meyer-FI	M	KCNT1	6	8	LOMBARDIA
598	Meyer-FI	M	KCNT1	23	30	LOMBARDIA
599	Meyer-FI	M	KCNT1	24	25	ABRUZZI
600	Meyer-FI	F	KCNT1	11	12	ABRUZZI
601	Meyer-FI	M	KCNT1	26	27	TOSCANA
602	Meyer-FI	F	MECP2	4	14	LOMBARDIA
603	Meyer-FI	F	MECP2	2	12	PUGLIE
604	Meyer-FI	F	MECP2	3	13	SICILIA
605	Meyer-FI	F	MECP2	0	10	TOSCANA
606	Meyer-FI	F	MECP2	8	16	.
607	Meyer-FI	F	MECP2	2	10	LAZIO
608	Meyer-FI	F	MECP2	4	11	EMILIA ROMAGNA
609	Meyer-FI	F	MECP2	4	10	PUGLIE
610	Meyer-FI	F	MECP2	2	8	TOSCANA
611	Meyer-FI	F	MECP2	3	10	ABRUZZI
612	Meyer-FI	F	MECP2	5	12	EMILIA ROMAGNA
613	Meyer-FI	F	MECP2	2	10	TOSCANA
614	Meyer-FI	F	MECP2	6	13	PIEMONTE
615	Meyer-FI	F	MECP2	21	27	CAMPANIA
616	Meyer-FI	F	MECP2	19	26	TOSCANA
617	Meyer-FI	F	MECP2	6	12	CALABRIA
618	Meyer-FI	F	MECP2	14	15	UMBRIA
619	Meyer-FI	M	MECP2	8	12	MARCHE
620	Meyer-FI	M	MECP2	10	15	LIGURIA
621	Meyer-FI	F	MECP2	3	8	TRENTINO A. A.
622	Meyer-FI	F	MECP2	2	6	.
623	Meyer-FI	F	MECP2	3	7	LAZIO

624	Meyer-FI	M	MECP2	5	9	CAMPANIA
625	Meyer-FI	F	MECP2	2	6	PUGLIE
626	Meyer-FI	F	MECP2	4	8	LOMBARDIA
627	Meyer-FI	F	MECP2	4	7	VENETO
628	Meyer-FI	F	MECP2	8	11	TOSCANA
629	Meyer-FI	M	MECP2	4	7	SICILIA
630	Meyer-FI	F	MECP2	2	5	LAZIO
631	Meyer-FI	M	MECP2	23	26	.
632	Meyer-FI	M	MECP2	30	32	TOSCANA
633	Meyer-FI	F	MECP2	11	13	VENETO
634	Meyer-FI	F	MECP2	12	13	CAMPANIA
635	Meyer-FI	F	MECP2	3	4	VENETO
636	Meyer-FI	F	MECP2	5	5	TOSCANA
637	Meyer-FI	F	MECP2	3	3	CAMPANIA
638	Meyer-FI	F	MECP2	5	5	FRIULI V. G.
639	Meyer-FI	F	MECP2	2	2	TOSCANA
640	Meyer-FI	F	MECP2	1	1	UMBRIA
641	Meyer-FI	M	MEF2C	2	5	LAZIO
642	Meyer-FI	F	NEXMIF	12	16	VENETO
643	Meyer-FI	F	NEXMIF	10	14	VENETO
644	Meyer-FI	F	NEXMIF	13	17	TOSCANA
645	Meyer-FI	F	NEXMIF	15	15	CAMPANIA
646	Meyer-FI	F	NEXMIF	20	20	TOSCANA
647	Meyer-FI	F	PCDH19	5	14	PUGLIE
648	Meyer-FI	F	PCDH19	13	22	TOSCANA
649	Meyer-FI	M	PCDH19	2	7	TOSCANA
650	Meyer-FI	F	PCDH19	4	8	TOSCANA
651	Meyer-FI	F	PCDH19	1	4	TOSCANA
652	Meyer-FI	F	PCDH19	13	19	EMILIA ROMAGNA
653	Meyer-FI	F	PCDH19	22	25	LOMBARDIA
654	Meyer-FI	F	PCDH19	8	11	SICILIA
655	Meyer-FI	F	PCDH19	1	2	LOMBARDIA
656	Meyer-FI	F	PCDH19	2	3	TOSCANA
657	Meyer-FI	F	PCDH19	8	9	CALABRIA
658	Meyer-FI	F	PCDH19	4	5	SICILIA
659	Meyer-FI	F	PCDH19	13	15	.
660	Meyer-FI	F	PCDH19	3	5	CAMPANIA
661	Meyer-FI	M	PIGA	12	18	TOSCANA
662	Meyer-FI	M	PIGA	8	8	TOSCANA
663	Meyer-FI	M	PIGA	2	4	BASILICATA
664	Meyer-FI	M	PIGA	4	5	.
665	Meyer-FI	M	PIGA	1	6	TOSCANA
666	Meyer-FI	M	RNF13	7	8	PIEMONTE
667	Meyer-FI	F	SCN1A	11	17	PUGLIE
668	Meyer-FI	M	SCN1A	6	12	.
669	Meyer-FI	F	SCN1A	1	7	TOSCANA
670	Meyer-FI	F	SCN1A	15	21	.
671	Meyer-FI	F	SCN1A	34	40	MARCHE
672	Meyer-FI	F	SCN1A	5	10	.
673	Meyer-FI	M	SCN1A	1	6	TRENTINO A. A.
674	Meyer-FI	F	SCN1A	1	6	CALABRIA
675	Meyer-FI	F	SCN1A	1	6	TOSCANA

676	Meyer-FI	F	SCN1A	23	27	.
677	Meyer-FI	F	SCN1A	21	25	EMILIA ROMAGNA
678	Meyer-FI	M	SCN1A	4	8	EMILIA ROMAGNA
679	Meyer-FI	F	SCN1A	1	5	TOSCANA
680	Meyer-FI	M	SCN1A	1	5	TOSCANA
681	Meyer-FI	M	SCN1A	3	7	TOSCANA
682	Meyer-FI	M	SCN1A	1	7	TOSCANA
683	Meyer-FI	M	SCN1A	1	5	UMBRIA
684	Meyer-FI	M	SCN1A	7	11	TRENTINO A. A.
685	Meyer-FI	F	SCN1A	2	5	TRENTINO A. A.
686	Meyer-FI	M	SCN1A	7	9	LOMBARDIA
687	Meyer-FI	F	SCN1A	31	34	EMILIA ROMAGNA
688	Meyer-FI	M	SCN1A	4	7	CAMPANIA
689	Meyer-FI	M	SCN1A	17	20	PUGLIE
690	Meyer-FI	M	SCN1A	47	50	ABRUZZI
691	Meyer-FI	M	SCN1A	1	4	MARCHE
692	Meyer-FI	M	SCN1A	2	4	CAMPANIA
693	Meyer-FI	M	BRAT1	3	6	SARDEGNA
694	Meyer-FI	F	SCN1A	0	2	PIEMONTE
695	Meyer-FI	F	CDKL5	1	5	LAZIO
696	Meyer-FI	F	SCN1A	0	2	PIEMONTE
697	Meyer-FI	F	SCN1A	21	22	PUGLIE
698	Meyer-FI	M	SCN1A	1	2	UMBRIA
699	Meyer-FI	M	SCN1A	1	2	TOSCANA
700	Meyer-FI	F	SCN1A	1	2	SICILIA
701	Meyer-FI	M	SCN1A	2	3	SICILIA
702	Meyer-FI	F	SCN1A	34	35	PIEMONTE
703	Meyer-FI	F	SCN1A	1	2	EMILIA ROMAGNA
704	Meyer-FI	F	SCN1A	10	10	.
705	Meyer-FI	M	SCN1A	2	2	PIEMONTE
706	Meyer-FI	M	SCN1A	1	1	UMBRIA
707	Meyer-FI	M	SCN1A	3	3	EMILIA ROMAGNA
708	Meyer-FI	F	SCN1A	0	0	EMILIA ROMAGNA
709	Meyer-FI	M	SCN1A	17	22	LOMBARDIA
710	Meyer-FI	F	SCN1A	8	16	LIGURIA
711	Meyer-FI	F	SCN1A	32	37	TOSCANA
712	Meyer-FI	M	SCN1A	3	10	EMILIA ROMAGNA
713	Meyer-FI	M	SCN1A	2	9	PUGLIE
714	Meyer-FI	F	SCN1A	13	18	PIEMONTE
715	Meyer-FI	F	SCN1A	24	29	PUGLIE
716	Meyer-FI	M	SCN1A	1	6	TOSCANA
717	Meyer-FI	F	SCN1A	4	7	PUGLIE
718	Meyer-FI	M	SCN1A	5	8	TOSCANA
719	Meyer-FI	M	SCN1A	21	23	EMILIA ROMAGNA
720	Meyer-FI	M	SCN1A	10	12	CALABRIA
721	Meyer-FI	F	SCN1A	14	16	VENETO
722	Meyer-FI	M	SCN2A	5	12	.
723	Meyer-FI	M	SCN2A	12	20	LOMBARDIA
724	Meyer-FI	M	SCN2A	2	9	.
725	Meyer-FI	M	SCN2A	3	10	EMILIA ROMAGNA
726	Meyer-FI	M	SCN2A	1	8	UMBRIA
727	Meyer-FI	M	SCN2A	3	10	LOMBARDIA

728	Meyer-FI	M	SCN2A	4	12	EMILIA ROMAGNA
729	Meyer-FI	F	SCN2A	1	7	LAZIO
730	Meyer-FI	F	SCN2A	8	14	LAZIO
731	Meyer-FI	F	SCN2A	1	11	UMBRIA
732	Meyer-FI	F	SCN2A	4	11	TRENTINO A. A.
733	Meyer-FI	M	SCN2A	4	10	CALABRIA
734	Meyer-FI	F	SCN2A	5	10	FRIULI V. G.
735	Meyer-FI	M	SCN2A	7	12	TOSCANA
736	Meyer-FI	F	SCN2A	12	17	LOMBARDIA
737	Meyer-FI	F	SCN2A	5	10	TOSCANA
738	Meyer-FI	M	SCN2A	14	19	LOMBARDIA
739	Meyer-FI	M	SCN2A	2	6	TOSCANA
740	Meyer-FI	F	SCN2A	1	5	SICILIA
741	Meyer-FI	F	SCN2A	15	18	LOMBARDIA
742	Meyer-FI	F	SCN2A	2	5	EMILIA ROMAGNA
743	Meyer-FI	F	SCN2A	17	20	LAZIO
744	Meyer-FI	F	SCN2A	12	15	LOMBARDIA
745	Meyer-FI	M	SCN2A	14	16	VENETO
746	Meyer-FI	M	SCN2A	2	4	EMILIA ROMAGNA
747	Meyer-FI	M	SCN2A	0	2	TOSCANA
748	Meyer-FI	M	SCN2A	4	6	LAZIO
749	Meyer-FI	F	SCN2A	6	7	LAZIO
750	Meyer-FI	M	SCN2A	19	20	ABRUZZI
751	Meyer-FI	F	SCN2A	16	17	CAMPANIA
752	Meyer-FI	F	SCN2A	0	1	LOMBARDIA
753	Meyer-FI	F	SCN2A	2	2	MARCHE
754	Meyer-FI	M	SCN2A	1	1	MOLISE
755	Meyer-FI	M	SCN2A	0	0	EMILIA ROMAGNA
756	Meyer-FI	F	SCN8A	2	9	EMILIA ROMAGNA
757	Meyer-FI	F	SCN8A	5	14	LAZIO
758	Meyer-FI	M	SCN8A	1	8	TOSCANA
759	Meyer-FI	F	SCN8A	12	18	LOMBARDIA
760	Meyer-FI	M	SCN8A	8	14	.
761	Meyer-FI	F	SCN8A	9	15	LOMBARDIA
762	Meyer-FI	M	SCN8A	2	7	LOMBARDIA
763	Meyer-FI	M	SCN8A	24	29	LOMBARDIA
764	Meyer-FI	M	SCN8A	13	18	MARCHE
765	Meyer-FI	F	SCN8A	4	10	LOMBARDIA
766	Meyer-FI	M	SCN8A	11	16	LAZIO
767	Meyer-FI	F	SCN8A	30	34	LOMBARDIA
768	Meyer-FI	F	SCN8A	5	9	UMBRIA
769	Meyer-FI	M	SCN8A	2	3	EMILIA ROMAGNA
770	Meyer-FI	F	SCN8A	0	2	TOSCANA
771	Meyer-FI	F	SCN8A	2	3	SICILIA
772	Meyer-FI	F	SCN8A	12	14	LAZIO
773	Meyer-FI	F	SCN8A	1	3	TOSCANA
774	Meyer-FI	F	SCN8A	2	5	LOMBARDIA
775	Meyer-FI	F	SCN8A	9	13	TOSCANA
776	Meyer-FI	M	SCN8A	8	11	.
777	Meyer-FI	F	SCN8A	11	14	CAMPANIA
778	Meyer-FI	F	SCN8A	18	18	ABRUZZI
779	Meyer-FI	M	SLC2A1	8	14	CALABRIA

780	Meyer-FI	M	SLC2A1	11	14	PUGLIE
781	Meyer-FI	F	SLC2A1	11	14	CAMPANIA
782	Meyer-FI	M	SLC2A1	12	16	SICILIA
783	Meyer-FI	F	SLC2A1	13	16	TRENTINO A. A.
784	Meyer-FI	M	SLC2A1	20	25	BASILICATA
785	Meyer-FI	F	SLC2A1	21	23	.
786	Meyer-FI	M	SLC2A1	16	26	EMILIA ROMAGNA
787	Meyer-FI	F	SLC2A1	18	27	EMILIA ROMAGNA
788	Meyer-FI	M	SLC2A1	4	12	PUGLIE
789	Meyer-FI	F	SLC2A1	5	11	CAMPANIA
790	Meyer-FI	F	SLC2A1	8	11	VENETO
791	Meyer-FI	F	SLC2A1	7	14	TOSCANA
792	Meyer-FI	F	SLC2A1	16	22	TOSCANA
793	Meyer-FI	F	SLC2A1	1	2	TOSCANA
794	Meyer-FI	F	SLC35A2	7	10	LOMBARDIA
795	Meyer-FI	M	SLC35A2	4	5	TRENTINO A. A.
796	Meyer-FI	M	SLC35A2	7	8	LAZIO
797	Meyer-FI	F	SLC6A1	13	18	TOSCANA
798	Meyer-FI	M	SLC6A1	16	21	PUGLIE
799	Meyer-FI	F	SLC6A1	8	13	PUGLIE
800	Meyer-FI	M	SLC6A1	5	9	LOMBARDIA
801	Meyer-FI	F	SLC6A1	10	13	LOMBARDIA
802	Meyer-FI	M	SLC6A1	14	18	TOSCANA
803	Meyer-FI	M	SLC6A1	13	16	TRENTINO A. A.
804	Meyer-FI	F	SLC6A1	7	9	CAMPANIA
805	Meyer-FI	M	SLC6A1	8	10	EMILIA ROMAGNA
806	Meyer-FI	M	SLC6A1	3	4	TOSCANA
807	Meyer-FI	F	SLC6A1	9	13	LOMBARDIA
808	Meyer-FI	M	SLC6A1	15	21	TOSCANA
809	Meyer-FI	M	SLC6A1	3	4	TOSCANA
810	Meyer-FI	F	GRIN1	0	3	EMILIA ROMAGNA
811	Meyer-FI	M	SLC6A1	9	9	LAZIO
812	Meyer-FI	F	SLC6A1	5	5	TOSCANA
813	Meyer-FI	M	SLC6A1	24	24	VENETO
814	Meyer-FI	M	SLC9A6	17	22	VENETO
815	Meyer-FI	M	SLC9A6	3	4	MARCHE
816	Meyer-FI	F	SLC9A6	5	10	PUGLIE
817	Meyer-FI	F	SMC1A	18	21	TOSCANA
818	Meyer-FI	F	SMC1A	15	18	TRENTINO A. A.
819	Meyer-FI	F	SMC1A	2	4	TOSCANA
820	Meyer-FI	F	SMC1A	6	6	.
821	Meyer-FI	M	SPTAN1	4	10	PUGLIE
822	Meyer-FI	M	SPTAN1	4	8	PUGLIE
823	Meyer-FI	M	SPTAN1	4	5	PUGLIE
824	Meyer-FI	F	SPTAN1	8	9	LOMBARDIA
825	Meyer-FI	M	STXBP1	1	6	EMILIA ROMAGNA
826	Meyer-FI	M	STXBP1	14	17	TOSCANA
827	Meyer-FI	M	STXBP1	7	11	PUGLIE
828	Meyer-FI	F	STXBP1	6	10	LAZIO
829	Meyer-FI	M	STXBP1	2	5	SICILIA
830	Meyer-FI	F	STXBP1	10	12	EMILIA ROMAGNA
831	Meyer-FI	F	STXBP1	9	10	LOMBARDIA

832	Meyer-FI	M	STXBP1	5	6	FRIULI V. G.
833	Meyer-FI	F	STXBP1	1	2	TOSCANA
834	Meyer-FI	F	STXBP1	2	9	PIEMONTE
835	Meyer-FI	F	STXBP1	2	9	TOSCANA
836	Meyer-FI	F	STXBP1	6	14	EMILIA ROMAGNA
837	Meyer-FI	F	STXBP1	2	11	.
838	Meyer-FI	M	STXBP1	1	10	TOSCANA
839	Meyer-FI	F	STXBP1	1	9	EMILIA ROMAGNA
840	Meyer-FI	F	STXBP1	10	19	TOSCANA
841	Meyer-FI	F	STXBP1	4	12	TRENTINO A. A.
842	Meyer-FI	M	STXBP1	10	15	LAZIO
843	Meyer-FI	M	STXBP1	9	12	.
844	Meyer-FI	M	STXBP1	3	9	PIEMONTE
845	Meyer-FI	F	SYNGAP1	20	25	VENETO
846	Meyer-FI	F	SYNGAP1	14	20	TOSCANA
847	Meyer-FI	M	SYNGAP1	10	16	TOSCANA
848	Meyer-FI	M	SYNGAP1	8	9	CALABRIA
849	Meyer-FI	M	SYNGAP1	4	9	UMBRIA
850	Meyer-FI	F	SYNGAP1	10	16	.
851	Meyer-FI	M	SYNGAP1	6	11	SICILIA
852	Meyer-FI	M	SYNGAP1	14	19	TOSCANA
853	Meyer-FI	M	SYNGAP1	5	10	EMILIA ROMAGNA
854	Meyer-FI	M	SYNGAP1	15	19	UMBRIA
855	Meyer-FI	M	SYNGAP1	12	15	TOSCANA
856	Meyer-FI	M	SYNGAP1	3	6	CAMPANIA
857	Meyer-FI	M	SYNGAP1	11	13	LOMBARDIA
858	Meyer-FI	F	SYNGAP1	3	4	TOSCANA
859	Meyer-FI	M	SYNGAP1	11	11	LAZIO
860	Meyer-FI	F	SYNGAP1	11	11	EMILIA ROMAGNA
861	Meyer-FI	F	TBC1D24	10	13	PIEMONTE
862	Meyer-FI	F	TBC1D24	5	9	LAZIO
863	Meyer-FI	F	TBC1D24	5	10	EMILIA ROMAGNA
864	Meyer-FI	M	TBC1D24	12	17	TOSCANA
865	Meyer-FI	M	YWHAG	15	17	MARCHE
866	Meyer-FI	M	YWHAG	6	8	TOSCANA
867	Meyer-FI	F	KCNQ2	1	2	LOMBARDIA
868	Meyer-FI	M	KCNT1	0	2	CALABRIA
869	Meyer-FI	F	ARHGEF9	5	7	CALABRIA
870	Meyer-FI	M	ARHGEF9	7	11	EMILIA ROMAGNA
871	Meyer-FI	M	ARV1	3	4	LOMBARDIA
872	Meyer-FI	F	CAD	3	4	TOSCANA
873	Meyer-FI	F	CLTC	4	9	VENETO
874	Meyer-FI	F	CLTC	0	0	TRENTINO A. A.
875	Meyer-FI	M	CSNK2B	17	19	TOSCANA
876	Meyer-FI	M	CSNK2B	3	4	TOSCANA
877	Meyer-FI	F	CSNK2B	13	15	TOSCANA
878	Meyer-FI	M	CSNK2B	2	4	LAZIO
879	Meyer-FI	M	CSNK2B	2	3	TOSCANA
880	Meyer-FI	F	CSNK2B	7	8	CALABRIA
881	Meyer-FI	F	CSNK2B	2	3	TOSCANA
882	Meyer-FI	M	CSNK2B	11	11	TOSCANA
883	Meyer-FI	F	CSNK2B	2	2	SICILIA

884	Meyer-FI	M	CYFIP2	2	4	TOSCANA
885	Meyer-FI	F	DHDDS	16	20	TOSCANA
886	Meyer-FI	F	GRIN2B	23	28	EMILIA ROMAGNA
887	Meyer-FI	F	GRIN1	1	1	SARDEGNA
888	Meyer-FI	F	HNRNPU	26	29	CAMPANIA
889	Meyer-FI	F	HNRNPU	24	28	LOMBARDIA
890	Meyer-FI	F	KMT2E	8	11	EMILIA ROMAGNA
891	Meyer-FI	M	KMT2E	23	23	BASILICATA
892	Meyer-FI	M	KMT2E	5	5	PIEMONTE
893	Meyer-FI	M	KMT2E	16	18	EMILIA ROMAGNA
894	Meyer-FI	F	KMT2E	10	10	TOSCANA
895	Meyer-FI	F	KMT2E	3	3	LAZIO
896	Meyer-FI	M	MBD5	31	33	TOSCANA
897	Meyer-FI	F	PACS2	6	10	TOSCANA
898	Meyer-FI	F	PACS2	5	6	PIEMONTE
899	Meyer-FI	F	PARS2	0	0	TOSCANA
900	Meyer-FI	F	PNKP	12	15	TOSCANA
901	Meyer-FI	F	PNKP	18	18	LAZIO
902	Meyer-FI	M	PNKP	6	6	MARCHE
903	Meyer-FI	M	PNKP	22	26	LOMBARDIA
904	Meyer-FI	F	PNKP	16	18	LIGURIA
905	Meyer-FI	M	PNKP	8	10	ABRUZZI
906	Meyer-FI	F	PNKP	29	31	EMILIA ROMAGNA
907	Meyer-FI	M	PNKP	1	3	TRENTINO A. A.
908	Meyer-FI	M	PNPO	7	15	PIEMONTE
909	Meyer-FI	F	PCDH19	18	26	LOMBARDIA
910	Meyer-FI	M	PNPO	0	5	EMILIA ROMAGNA
911	Meyer-FI	F	POLG	0	1	TOSCANA
912	Meyer-FI	F	POLG	17	20	CAMPANIA
913	Meyer-FI	M	POLG	1	2	TRENTINO A. A.
914	Meyer-FI	F	PURA	9	15	CAMPANIA
915	Meyer-FI	M	PURA	10	15	.
916	Meyer-FI	F	PURA	2	6	.
917	Meyer-FI	F	PURA	17	21	LOMBARDIA
918	Meyer-FI	F	PURA	18	22	LOMBARDIA
919	Meyer-FI	M	PURA	14	16	TOSCANA
920	Meyer-FI	M	PURA	6	6	PIEMONTE
921	Meyer-FI	M	SCN1B	9	9	UMBRIA
922	Meyer-FI	F	SCN1B	10	11	SICILIA
923	Meyer-FI	M	SCN1B	5	9	LAZIO
924	Meyer-FI	M	SCN1B	11	13	TOSCANA
925	Meyer-FI	M	SCN1B	14	17	LAZIO
926	Meyer-FI	F	SCN1B	1	1	EMILIA ROMAGNA
927	Meyer-FI	F	SCN1B	13	19	TOSCANA
928	Meyer-FI	M	SCN3A	2	4	EMILIA ROMAGNA
929	Meyer-FI	F	SCN3A	1	5	TOSCANA
930	Meyer-FI	M	SLC1A2	5	5	TOSCANA
931	Meyer-FI	M	SLC13A5	1	5	CAMPANIA
932	Meyer-FI	F	SLC13A5	10	14	LAZIO
933	Meyer-FI	F	ST3GAL5	5	9	PUGLIE
934	Meyer-FI	M	SZT2	18	23	TRENTINO A. A.
935	Meyer-FI	F	SZT2	4	10	.

936	Meyer-FI	F	WWOX	1	5	TOSCANA
937	Meyer-FI	F	WWOX	25	26	BASILICATA
938	Mondino-Pavia	M	BRAT1	10	10	CAMPANIA
939	Mondino-Pavia	F	MECP2	3	4	ABRUZZI
940	Mondino-Pavia	F	SPTAN1	0	1	EMILIA ROMAGNA
941	Mondino-Pavia	F	SCN1A	1	1	TRENTINO A. A.
942	Mondino-Pavia	F	SZT2	1	1	LOMBARDIA
943	Mondino-Pavia	F	ALG13	8	8	.
944	Mondino-Pavia	M	ATP6V1A	9	10	LOMBARDIA
945	Mondino-Pavia	M	CACNA1A	11	11	LOMBARDIA
946	Mondino-Pavia	F	CACNA1A	15	15	PIEMONTE
947	Mondino-Pavia	F	CASK	13	15	PUGLIE
948	Mondino-Pavia	M	CASK	2	5	LOMBARDIA
949	Mondino-Pavia	F	CDKL5	0	0	LOMBARDIA
950	Mondino-Pavia	M	CDKL5	1	6	LOMBARDIA
951	Mondino-Pavia	M	CDKL5	1	5	PIEMONTE
952	Mondino-Pavia	M	CHD2	30	33	PIEMONTE
953	Mondino-Pavia	M	CHD2	19	20	LOMBARDIA
954	Mondino-Pavia	F	CHD2	20	20	LOMBARDIA
955	Mondino-Pavia	M	GABRB3	3	7	LOMBARDIA
956	Mondino-Pavia	F	GABRG2	0	3	LOMBARDIA
957	Mondino-Pavia	F	GABRG2	3	3	LOMBARDIA
958	Mondino-Pavia	F	GNAO1	19	22	PIEMONTE
959	Mondino-Pavia	M	GRIN2A	9	10	PIEMONTE
960	Mondino-Pavia	M	GRIN2B	17	19	LOMBARDIA
961	Mondino-Pavia	M	GRIN2B	13	13	CALABRIA
962	Mondino-Pavia	F	KCNA2	3	4	ABRUZZI
963	Mondino-Pavia	M	KCNA2	20	20	TRENTINO A. A.
964	Mondino-Pavia	M	KCNQ2	6	8	.
965	Mondino-Pavia	M	KCNQ2	10	14	LOMBARDIA
966	Mondino-Pavia	F	KCNT1	15	19	LOMBARDIA
967	Mondino-Pavia	M	KCNT1	37	39	PIEMONTE
968	Mondino-Pavia	F	KCNT1	7	11	EMILIA ROMAGNA
969	Mondino-Pavia	F	MECP2	5	9	LOMBARDIA
970	Mondino-Pavia	F	MECP2	4	6	LOMBARDIA
971	Mondino-Pavia	F	MECP2	2	2	ABRUZZI
972	Mondino-Pavia	F	MECP2	14	18	LOMBARDIA
973	Mondino-Pavia	F	PCDH19	4	4	TRENTINO A. A.
974	Mondino-Pavia	F	PCDH19	31	31	TRENTINO A. A.
975	Mondino-Pavia	F	PURA	13	15	.
976	Mondino-Pavia	F	PURA	3	3	LOMBARDIA
977	Mondino-Pavia	F	SCN1A	1	1	TRENTINO A. A.
978	Mondino-Pavia	M	SCN1A	29	29	CALABRIA
979	Mondino-Pavia	M	SCN1A	5	9	CAMPANIA
980	Mondino-Pavia	F	SCN8A	45	46	LOMBARDIA
981	Mondino-Pavia	F	SCN8A	16	17	LOMBARDIA
982	Mondino-Pavia	F	SCN8A	5	9	UMBRIA
983	Mondino-Pavia	F	SLC2A1	29	31	.
984	Mondino-Pavia	F	SLC2A1	11	14	LOMBARDIA
985	Mondino-Pavia	M	SLC6A1	20	21	TRENTINO A. A.
986	Mondino-Pavia	M	SLC6A1	21	21	LOMBARDIA
987	Mondino-Pavia	F	SLC6A1	6	8	LOMBARDIA

**988**	Mondino-Pavia	F	SLC6A1	3	3	PIEMONTE
**989**	Mondino-Pavia	M	SLC9A6	8	9	VENETO
**990**	Mondino-Pavia	M	SPTAN1	16	19	LOMBARDIA
**991**	Mondino-Pavia	F	SPTAN1	13	16	LOMBARDIA
**992**	Mondino-Pavia	M	STXBP1	28	29	EMILIA ROMAGNA
**993**	Mondino-Pavia	F	STXBP1	17	17	LOMBARDIA
**994**	Mondino-Pavia	F	SYNGAP1	3	3	LOMBARDIA
**995**	Mondino-Pavia	M	SYNGAP1	21	21	.
**996**	Mondino-Pavia	M	ATP1A2	12	14	PUGLIE
**997**	Mondino-Pavia	M	CACNA1A	43	45	.
**998**	Mondino-Pavia	F	CACNA1A	19	20	.
**999**	Mondino-Pavia	F	CACNA1A	52	53	.
**1000**	Mondino-Pavia	M	CACNA1A	34	35	.
**1001**	Mondino-Pavia	M	CACNA1A	58	59	LOMBARDIA
**1002**	Mondino-Pavia	F	CASK	15	16	LOMBARDIA
**1003**	Mondino-Pavia	F	CASK	2	2	LOMBARDIA
**1004**	Mondino-Pavia	F	CASK	5	5	LOMBARDIA
**1005**	Mondino-Pavia	F	CLTC	3	3	LOMBARDIA
**1006**	Mondino-Pavia	M	GNAO1	9	10	LIGURIA
**1007**	Mondino-Pavia	M	HCN1	6	7	LOMBARDIA
**1008**	Mondino-Pavia	F	MECP2	4	7	LOMBARDIA
**1009**	Mondino-Pavia	F	MECP2	9	11	.
**1010**	Mondino-Pavia	F	SYNGAP1	1	1	TRENTINO A. A.
**1011**	NeuroPedAOB	F	SPTAN1	4	9	SARDEGNA
**1012**	NeuroPedAOB	M	GRIN2B	7	8	SARDEGNA
**1013**	NeuroPedAOB	F	HCN1	2	4	SARDEGNA
**1014**	NeuroPedAOB	F	SCN1A	2	3	SARDEGNA
**1015**	NeuroPedAOB	M	SCN1A	1	8	SARDEGNA
**1016**	NeuroPedAOB	F	SCN2A	6	9	SARDEGNA
**1017**	NeuroPedAOB	F	GRIN2A	10	10	SARDEGNA
**1018**	NeuroPedAOB	M	PIGA	5	5	SARDEGNA
**1019**	NeuroPedAOB	F	GRIN2B	2	6	SARDEGNA
**1020**	NeuroPedAOB	M	GABRB3	10	14	SARDEGNA
**1021**	NeuroPedAOB	F	DNM1	7	12	SARDEGNA
**1022**	NeuroPedAOB	F	SCN1A	5	15	SARDEGNA
**1023**	NeuroPedAOB	F	SCN1A	4	4	SARDEGNA
**1024**	NeuroPedAOB	F	SCN1A	1	11	SARDEGNA
**1025**	NeuroPedAOB	M	SCN1A	1	10	SARDEGNA
**1026**	NeuroPedAOB	M	PURA	25	28	SARDEGNA
**1027**	NPIv.Sabelli-RM	M	CHD2	20	22	LAZIO
**1028**	NPIv.Sabelli-RM	M	SCN1A	15	22	LAZIO
**1029**	NPIv.Sabelli-RM	M	PIGQ	17	21	LAZIO
**1030**	NPIv.Sabelli-RM	F	SLC6A1	18	21	LAZIO
**1031**	NPIv.Sabelli-RM	F	CDKL5	15	20	LAZIO
**1032**	NPIv.Sabelli-RM	F	CHD2	12	19	LAZIO
**1033**	NPIv.Sabelli-RM	F	SYNGAP1	16	18	LAZIO
**1034**	NPIv.Sabelli-RM	M	GABRG2	17	17	LAZIO
**1035**	NPIv.Sabelli-RM	F	SLC2A1	12	16	LAZIO
**1036**	NPIv.Sabelli-RM	M	SCN8A	15	16	LAZIO
**1037**	NPIv.Sabelli-RM	F	PURA	11	16	LAZIO
**1038**	NPIv.Sabelli-RM	M	ATP1A3	8	14	LAZIO
**1039**	NPIv.Sabelli-RM	F	SCN1A	10	14	LAZIO

1040	NPIv.Sabelli-RM	M	SYNGAP1	10	13	.
1041	NPIv.Sabelli-RM	F	SLC2A1	4	14	EMILIA ROMAGNA
1042	NPIv.Sabelli-RM	F	CACNA1E	1	3	LAZIO
1043	NPIv.Sabelli-RM	F	STXBP1	1	3	CAMPANIA
1044	NPIv.Sabelli-RM	F	GRIN2A	21	23	LAZIO
1045	NPIv.Sabelli-RM	F	GRIN2A	18	20	LAZIO
1046	NPIv.Sabelli-RM	F	MECP2	4	9	ABRUZZI
1047	NPIv.Sabelli-RM	M	SLC2A1	9	15	.
1048	NPIv.Sabelli-RM	F	SCN2A	6	11	LAZIO
1049	NPIv.Sabelli-RM	F	CDKL5	2	4	LAZIO
1050	NPIv.Sabelli-RM	M	GRIN1	12	17	.
1051	NPIv.Sabelli-RM	F	CHD2	8	12	LAZIO
1052	NPIv.Sabelli-RM	M	KCNQ2	3	7	LAZIO
1053	NPIv.Sabelli-RM	F	SCN1A	6	9	LAZIO
1054	NPIv.Sabelli-RM	M	GNAO1	3	11	LAZIO
1055	NPIv.Sabelli-RM	M	DNM1	4	6	LAZIO
1056	NPIv.Sabelli-RM	M	SLC2A1	13	13	LAZIO
1057	NPIv.Sabelli-RM	M	SCN8A	16	16	LAZIO
1058	NPIv.Sabelli-RM	M	STXBP1	0	0	LAZIO
1059	NPIv.Sabelli-RM	M	SCN1A	9	9	LAZIO
1060	NPIv.Sabelli-RM	F	GABRB3	6	12	LAZIO
1061	NPIv.Sabelli-RM	M	SCN8A	13	14	LAZIO
1062	NPIv.Sabelli-RM	F	GNAO1	21	24	ABRUZZI
1063	NPIv.Sabelli-RM	M	GNAO1	4	6	LAZIO
1064	NPIv.Sabelli-RM	M	ATP1A3	21	24	LAZIO
1065	NPIv.Sabelli-RM	M	KCNA2	22	27	LAZIO
1066	NPIv.Sabelli-RM	F	BRAT1	20	23	LAZIO
1067	NPIv.Sabelli-RM	M	GNAO1	6	12	UMBRIA
1068	NPIv.Sabelli-RM	M	SLC2A1	9	11	LAZIO
1069	NPIv.Sabelli-RM	M	GRIN2A	7	11	LAZIO
1070	NPIv.Sabelli-RM	M	CACNA1A	6	10	PUGLIE
1071	NPIv.Sabelli-RM	F	SYNGAP1	3	8	LAZIO
1072	NPIv.Sabelli-RM	M	GNAO1	3	7	LAZIO
1073	NPIv.Sabelli-RM	F	FOXG1	1	6	LAZIO
1074	NPIv.Sabelli-RM	M	KCNT1	5	5	LAZIO
1075	NPIv.Sabelli-RM	M	PNPO	2	10	LAZIO
1076	NPIv.Sabelli-RM	F	SLC13A5	15	17	EMILIA ROMAGNA
1077	NPIv.Sabelli-RM	F	KCNQ2	29	32	LAZIO
1078	NPIv.Sabelli-RM	M	DHDDS	35	40	LAZIO
1079	NPIv.Sabelli-RM	M	DHDDS	9	14	SICILIA
1080	NPIv.Sabelli-RM	M	ARHGEF9	23	29	LAZIO
1081	Oasi-Troina	M	CACNA1A	19	20	SICILIA
1082	Oasi-Troina	F	CACNA1A	16	18	SICILIA
1083	Oasi-Troina	F	SCN1A	20	23	SICILIA
1084	Oasi-Troina	M	WWOX	10	12	SICILIA
1085	Oasi-Troina	M	SLC13A5	7	10	SICILIA
1086	Oasi-Troina	M	SLC13A5	13	16	SICILIA
1087	Oasi-Troina	M	KCNT1	33	34	SICILIA
1088	Oasi-Troina	F	KCNT1	25	26	SICILIA
1089	Oasi-Troina	F	CHD2	31	31	SICILIA
1090	Oasi-Troina	F	SMC1A	12	12	SICILIA
1091	Oasi-Troina	F	MECP2	5	12	SICILIA

**1092**	Oasi-Troina	F	SCN1A	40	41	SICILIA
**1093**	Oasi-Troina	M	TBC1D24	0	4	SICILIA
**1094**	Oasi-Troina	M	TBC1D24	8	12	SICILIA
**1095**	Oasi-Troina	F	MECP2	5	8	SICILIA
**1096**	Oasi-Troina	F	PCDH19	2	2	SICILIA
**1097**	Oasi-Troina	F	SCN2A	16	16	SICILIA
**1098**	Oasi-Troina	M	GABRB3	42	43	SICILIA
**1099**	Oasi-Troina	F	STXBP1	19	26	SICILIA
**1100**	OBG-RM	F	SCN1A	13	21	PUGLIE
**1101**	OBG-RM	F	SCN1A	3	12	LAZIO
**1102**	OBG-RM	F	SCN1A	8	17	LAZIO
**1103**	OBG-RM	F	SCN1A	9	18	LIGURIA
**1104**	OBG-RM	M	SCN1A	1	10	CAMPANIA
**1105**	OBG-RM	M	SCN1A	7	16	PUGLIE
**1106**	OBG-RM	M	SCN1A	2	11	LAZIO
**1107**	OBG-RM	F	PCDH19	2	11	BASILICATA
**1108**	OBG-RM	F	PCDH19	4	13	LAZIO
**1109**	OBG-RM	F	PCDH19	22	31	LAZIO
**1110**	OBG-RM	F	SCN1A	7	15	LAZIO
**1111**	OBG-RM	M	SCN1A	9	17	LAZIO
**1112**	OBG-RM	F	SCN1A	7	15	LAZIO
**1113**	OBG-RM	F	SCN1A	8	16	LAZIO
**1114**	OBG-RM	M	SCN1A	12	20	CALABRIA
**1115**	OBG-RM	F	SCN1A	5	13	CAMPANIA
**1116**	OBG-RM	M	SCN1A	9	17	PUGLIE
**1117**	OBG-RM	M	SCN1A	4	12	VENETO
**1118**	OBG-RM	F	SCN1A	4	12	CALABRIA
**1119**	OBG-RM	M	SCN1A	9	17	LAZIO
**1120**	OBG-RM	F	SCN1A	3	11	PUGLIE
**1121**	OBG-RM	M	SCN1A	6	14	CAMPANIA
**1122**	OBG-RM	M	SCN1A	23	31	CAMPANIA
**1123**	OBG-RM	M	SCN1A	27	35	PUGLIE
**1124**	OBG-RM	M	SCN1A	11	19	PUGLIE
**1125**	OBG-RM	M	SCN1A	18	26	CALABRIA
**1126**	OBG-RM	F	PCDH19	5	13	LAZIO
**1127**	OBG-RM	F	PCDH19	19	27	.
**1128**	OBG-RM	F	PCDH19	22	30	LAZIO
**1129**	OBG-RM	F	PCDH19	39	47	LAZIO
**1130**	OBG-RM	F	PCDH19	5	13	PUGLIE
**1131**	OBG-RM	F	PCDH19	7	15	LAZIO
**1132**	OBG-RM	F	PCDH19	12	20	UMBRIA
**1133**	OBG-RM	M	PCDH19	4	12	LAZIO
**1134**	OBG-RM	F	SCN1A	1	9	CAMPANIA
**1135**	OBG-RM	F	SCN1A	17	25	LAZIO
**1136**	OBG-RM	M	ATP1A3	1	9	LAZIO
**1137**	OBG-RM	M	CHD2	5	12	LAZIO
**1138**	OBG-RM	F	PCDH19	3	10	LAZIO
**1139**	OBG-RM	F	PCDH19	4	11	CALABRIA
**1140**	OBG-RM	M	PCDH19	4	11	CALABRIA
**1141**	OBG-RM	M	ATP1A2	7	14	CAMPANIA
**1142**	OBG-RM	M	CACNA1A	9	16	LAZIO
**1143**	OBG-RM	M	KCNB1	5	11	CAMPANIA

**1144**	OBG-RM	M	SCN8A	1	7	LAZIO
**1145**	OBG-RM	F	STXBP1	7	13	SICILIA
**1146**	OBG-RM	M	MEF2C	4	10	EMILIA ROMAGNA
**1147**	OBG-RM	M	CHD2	23	29	CAMPANIA
**1148**	OBG-RM	F	ALG13	2	8	LAZIO
**1149**	OBG-RM	M	SCN1A	2	8	LAZIO
**1150**	OBG-RM	M	SCN1A	5	11	PUGLIE
**1151**	OBG-RM	F	SCN8A	0	6	PUGLIE
**1152**	OBG-RM	M	SCN8A	9	15	CAMPANIA
**1153**	OBG-RM	F	CDKL5	2	8	LAZIO
**1154**	OBG-RM	M	GRIN2A	19	25	ABRUZZI
**1155**	OBG-RM	F	GRIN2A	33	39	ABRUZZI
**1156**	OBG-RM	F	SCN1A	9	15	SICILIA
**1157**	OBG-RM	F	PCDH19	14	20	PUGLIE
**1158**	OBG-RM	F	PCDH19	9	15	PUGLIE
**1159**	OBG-RM	F	CDKL5	16	22	CALABRIA
**1160**	OBG-RM	M	SCN1A	3	9	CAMPANIA
**1161**	OBG-RM	M	SCN1A	35	41	.
**1162**	OBG-RM	F	SCN1A	3	9	LAZIO
**1163**	OBG-RM	M	PIGA	1	6	CALABRIA
**1164**	OBG-RM	F	KCNQ2	2	7	CAMPANIA
**1165**	OBG-RM	F	GABRA1	4	9	ABRUZZI
**1166**	OBG-RM	F	KCNB1	30	35	LAZIO
**1167**	OBG-RM	M	STXBP1	1	6	LAZIO
**1168**	OBG-RM	F	BRAT1	2	7	LAZIO
**1169**	OBG-RM	M	MECP2	8	13	LAZIO
**1170**	OBG-RM	F	SCN1A	2	7	.
**1171**	OBG-RM	F	SCN1A	1	6	LAZIO
**1172**	OBG-RM	F	KCNQ2	0	5	LAZIO
**1173**	OBG-RM	F	KCNQ2	1	6	ABRUZZI
**1174**	OBG-RM	F	MECP2	3	8	LAZIO
**1175**	OBG-RM	F	CDKL5	0	5	CALABRIA
**1176**	OBG-RM	F	SCN1A	1	6	BASILICATA
**1177**	OBG-RM	M	KCNB1	3	8	LAZIO
**1178**	OBG-RM	F	SCN1A	1	6	LAZIO
**1179**	OBG-RM	F	MECP2	4	9	.
**1180**	OBG-RM	F	GRIN2B	9	14	ABRUZZI
**1181**	OBG-RM	F	SCN1A	4	9	BASILICATA
**1182**	OBG-RM	F	ATP1A2	29	34	LAZIO
**1183**	OBG-RM	M	FGF12	5	9	CAMPANIA
**1184**	OBG-RM	F	SYNGAP1	3	7	CAMPANIA
**1185**	OBG-RM	F	SLC2A1	26	30	LAZIO
**1186**	OBG-RM	F	ATP1A2	10	14	PUGLIE
**1187**	OBG-RM	F	SCN1A	14	18	MOLISE
**1188**	OBG-RM	F	SCN1A	14	18	MOLISE
**1189**	OBG-RM	F	SYNGAP1	4	8	LAZIO
**1190**	OBG-RM	F	SLC2A1	1	5	CAMPANIA
**1191**	OBG-RM	F	KCNT1	0	4	SICILIA
**1192**	OBG-RM	M	STXBP1	0	4	CALABRIA
**1193**	OBG-RM	F	CDKL5	9	13	.
**1194**	OBG-RM	M	GRIN2A	0	4	SICILIA
**1195**	OBG-RM	F	SCN8A	0	4	LAZIO

**1196**	OBG-RM	F	SCN2A	0	4	ABRUZZI
**1197**	OBG-RM	M	BRAT1	4	8	BASILICATA
**1198**	OBG-RM	F	CDKL5	2	6	CAMPANIA
**1199**	OBG-RM	F	DNM1	15	19	ABRUZZI
**1200**	OBG-RM	M	CHD2	7	11	LAZIO
**1201**	OBG-RM	F	STXBP1	5	9	PUGLIE
**1202**	OBG-RM	M	SCN2A	19	23	MARCHE
**1203**	OBG-RM	M	GABRB3	19	23	LAZIO
**1204**	OBG-RM	F	CDKL5	7	11	.
**1205**	OBG-RM	M	PIGA	15	19	.
**1206**	OBG-RM	M	ATP1A2	25	29	LAZIO
**1207**	OBG-RM	F	CACNA1A	2	6	LAZIO
**1208**	OBG-RM	M	GNAO1	18	22	EMILIA ROMAGNA
**1209**	OBG-RM	M	KCNQ2	1	4	CAMPANIA
**1210**	OBG-RM	F	SCN1A	1	4	PUGLIE
**1211**	OBG-RM	F	PCDH19	4	7	CAMPANIA
**1212**	OBG-RM	M	GRIN2A	5	8	CAMPANIA
**1213**	OBG-RM	F	STXBP1	14	17	CAMPANIA
**1214**	OBG-RM	F	CDKL5	26	29	LAZIO
**1215**	OBG-RM	F	PCDH19	2	5	LAZIO
**1216**	OBG-RM	F	GABRB3	1	4	BASILICATA
**1217**	OBG-RM	F	KCNQ2	19	22	LAZIO
**1218**	OBG-RM	M	SYNGAP1	7	10	CAMPANIA
**1219**	OBG-RM	F	GABRB3	1	4	LAZIO
**1220**	OBG-RM	F	SCN2A	38	41	LAZIO
**1221**	OBG-RM	F	GRIN2B	3	6	LAZIO
**1222**	OBG-RM	M	SPTAN1	15	18	LAZIO
**1223**	OBG-RM	M	GRIN2D	19	22	CAMPANIA
**1224**	OBG-RM	F	SCN1A	16	19	LAZIO
**1225**	OBG-RM	F	SLC2A1	16	19	CAMPANIA
**1226**	OBG-RM	F	SCN1A	20	23	.
**1227**	OBG-RM	F	SCN2A	5	8	.
**1228**	OBG-RM	F	GNAO1	15	18	CAMPANIA
**1229**	OBG-RM	F	CHD2	5	7	BASILICATA
**1230**	OBG-RM	F	CDKL5	1	3	ABRUZZI
**1231**	OBG-RM	F	SCN8A	16	18	CAMPANIA
**1232**	OBG-RM	F	SLC6A1	4	6	LAZIO
**1233**	OBG-RM	F	PCDH19	2	4	.
**1234**	OBG-RM	F	KCNB1	16	18	LAZIO
**1235**	OBG-RM	F	STXBP1	0	2	BASILICATA
**1236**	OBG-RM	M	KCNB1	2	4	LAZIO
**1237**	OBG-RM	M	GRIN2A	6	8	LAZIO
**1238**	OBG-RM	M	GABRB3	0	2	CALABRIA
**1239**	OBG-RM	F	CHD2	7	9	.
**1240**	OBG-RM	M	STXBP1	3	5	LAZIO
**1241**	OBG-RM	M	SCN2A	0	2	SICILIA
**1242**	OBG-RM	M	CHD2	24	26	LAZIO
**1243**	OBG-RM	M	KCNB1	12	14	LAZIO
**1244**	OBG-RM	M	GRIN1	4	6	LAZIO
**1245**	OBG-RM	F	GABRA1	22	24	LAZIO
**1246**	OBG-RM	M	SCN8A	17	19	PUGLIE
**1247**	OBG-RM	F	KCNQ2	12	14	SICILIA

1248	OBG-RM	F	GABRA1	14	16	LAZIO
1249	OBG-RM	M	SLC2A1	2	4	TOSCANA
1250	OBG-RM	M	GABRB2	8	10	LAZIO
1251	OBG-RM	M	SCN1A	1	3	LAZIO
1252	OBG-RM	M	ATP1A3	5	7	LAZIO
1253	OBG-RM	M	EEF1A2	4	5	PUGLIE
1254	OBG-RM	M	STXBP1	1	2	CAMPANIA
1255	OBG-RM	M	KCNQ2	3	4	.
1256	OBG-RM	M	SLC6A1	17	18	LAZIO
1257	OBG-RM	M	SCN8A	7	8	CAMPANIA
1258	OBG-RM	M	GABRA1	13	14	LAZIO
1259	OBG-RM	F	MECP2	7	8	LAZIO
1260	OBG-RM	M	SCN1A	1	2	SARDEGNA
1261	OBG-RM	F	PCDH19	2	3	LAZIO
1262	OBG-RM	M	KCNA2	6	7	LAZIO
1263	OBG-RM	M	SCN2A	31	32	CAMPANIA
1264	OBG-RM	F	PCDH19	1	2	BASILICATA
1265	OBG-RM	F	SCN1A	0	1	LAZIO
1266	OBG-RM	F	CHD2	0	1	LAZIO
1267	OBG-RM	F	ALG13	0	1	LAZIO
1268	OBG-RM	F	KCNQ2	12	13	CALABRIA
1269	OBG-RM	F	MECP2	4	5	LAZIO
1270	OBG-RM	F	CHD2	9	10	LAZIO
1271	OBG-RM	F	GABRG2	5	6	UMBRIA
1272	OBG-RM	F	MECP2	11	12	LAZIO
1273	OBG-RM	F	CASK	1	2	LAZIO
1274	OBG-RM	M	GABRB3	25	25	CAMPANIA
1275	OBG-RM	F	KCNQ2	1	1	LAZIO
1276	OBG-RM	M	EEF1A2	6	6	CAMPANIA
1277	OBG-RM	F	SCN1A	12	12	LAZIO
1278	OBG-RM	F	SCN8A	9	9	LAZIO
1279	OBG-RM	F	SYNGAP1	9	9	ABRUZZI
1280	OBG-RM	M	SCN8A	24	24	LAZIO
1281	OBG-RM	F	SCN8A	7	7	VENETO
1282	OBG-RM	F	NEXMIF	6	6	LAZIO
1283	OBG-RM	M	SCN1A	2	2	CAMPANIA
1284	OBG-RM	M	CACNA1A	4	4	CALABRIA
1285	OBG-RM	F	SYNGAP1	21	21	LAZIO
1286	OBG-RM	M	CDKL5	1	1	.
1287	OBG-RM	M	CLCN4	8	8	LAZIO
1288	OBG-RM	M	KCNB1	9	9	ABRUZZI
1289	OBG-RM	M	SCN1A	9	9	MARCHE
1290	OBG-RM	M	DNM1L	2	2	BASILICATA
1291	OBG-RM	M	SCN1A	1	1	LAZIO
1292	OBG-RM	M	GRIN1	10	12	LAZIO
1293	OBG-RM	F	GRIN2D	10	12	LAZIO
1294	OBG-RM	M	GNAO1	4	10	UMBRIA
1295	OBG-RM	M	KCNQ2	2	4	CALABRIA
1296	OBG-RM	F	MECP2	3	3	EMILIA ROMAGNA
1297	OBG-RM	F	WWOX	7	9	LAZIO
1298	OBG-RM	M	WWOX	21	24	CAMPANIA
1299	OBG-RM	M	WWOX	10	12	SICILIA

**1300**	OBG-RM	F	CDK19	1	3	.
**1301**	OBG-RM	M	CSNK2B	9	9	CALABRIA
**1302**	OBG-RM	M	CSNK2B	34	34	CALABRIA
**1303**	OBG-RM	F	CSNK2B	9	9	SICILIA
**1304**	OBG-RM	F	CSNK2B	2	2	CAMPANIA
**1305**	OBG-RM	F	CSNK2B	8	10	ABRUZZI
**1306**	OBG-RM	F	CSNK2B	17	18	CALABRIA
**1307**	OBG-RM	F	CSNK2B	11	12	CALABRIA
**1308**	OBG-RM	F	CSNK2B	40	41	CALABRIA
**1309**	OBG-RM	M	GRIN1	64	66	ABRUZZI
**1310**	OBG-RM	F	SZT2	4	7	LAZIO
**1311**	OBG-RM	F	SZT2	10	13	PUGLIE
**1312**	OBG-RM	F	SZT2	6	9	LAZIO
**1313**	OBG-RM	M	SZT2	1	4	CAMPANIA
**1314**	OBG-RM	F	SZT2	5	7	CAMPANIA
**1315**	OBG-RM	F	CYFIP2	0	0	CAMPANIA
**1316**	OBG-RM	F	FRRS1L	5	6	BASILICATA
**1317**	OBG-RM	M	FRRS1L	5	5	VENETO
**1318**	OBG-RM	F	GABRA5	2	3	PUGLIE
**1319**	OBG-RM	M	KMT2E	2	2	CALABRIA
**1320**	OBG-RM	F	MBD5	14	14	.
**1321**	OBG-RM	F	MBD5	45	45	LAZIO
**1322**	OBG-RM	M	PACS2	7	11	PUGLIE
**1323**	OBG-RM	M	PARS2	3	3	ABRUZZI
**1324**	OBG-RM	M	PNKP	1	3	.
**1325**	OBG-RM	F	SCN1B	4	6	LAZIO
**1326**	OBG-RM	M	POLG	1	4	CALABRIA
**1327**	OBG-RM	M	SCN1B	10	13	CAMPANIA
**1328**	OBG-RM	F	SCN1B	12	14	LAZIO
**1329**	OBG-RM	F	SLC13A5	24	27	CALABRIA
**1330**	OBG-RM	F	SLC13A5	24	27	CALABRIA
**1331**	OBG-RM	M	SLC1A2	16	18	SICILIA
**1332**	OBG-RM	F	ST3GAL3	14	15	LAZIO
**1333**	OBG-RM	F	KMT2E	7	7	.
**1334**	OBG-RM	F	SCN1B	4	7	CALABRIA
**1335**	OBG-RM	M	SCN1B	13	15	LOMBARDIA
**1336**	OBG-RM	M	SCN1A	23	31	LAZIO
**1337**	Salesi-Ancona	M	SCN2A	16	19	ABRUZZI
**1338**	Salesi-Ancona	F	SCN1A	15	19	ABRUZZI
**1339**	Salesi-Ancona	M	GABRG2	10	13	ABRUZZI
**1340**	Salesi-Ancona	F	SLC2A1	12	13	MARCHE
**1341**	Salesi-Ancona	M	SCN2A	11	12	ABRUZZI
**1342**	Salesi-Ancona	F	SCN1A	6	10	MARCHE
**1343**	Salesi-Ancona	F	KCNQ2	10	10	MARCHE
**1344**	Salesi-Ancona	F	SCN1A	2	10	MARCHE
**1345**	Salesi-Ancona	F	FOXG1	10	10	UMBRIA
**1346**	Salesi-Ancona	F	PCDH19	4	9	ABRUZZI
**1347**	Salesi-Ancona	F	SCN2A	1	6	ABRUZZI
**1348**	Salesi-Ancona	F	SLC2A1	6	8	EMILIA ROMAGNA
**1349**	Salesi-Ancona	F	CHD2	3	8	MARCHE
**1350**	Salesi-Ancona	M	SCN8A	1	7	MARCHE
**1351**	Salesi-Ancona	F	SCN1A	3	6	ABRUZZI

**1352**	Salesi-Ancona	F	STXBP1	0	5	MARCHE
**1353**	Salesi-Ancona	F	SCN8A	1	5	MARCHE
**1354**	Salesi-Ancona	F	SCN2A	0	4	MARCHE
**1355**	Salesi-Ancona	F	CDKL5	0	3	MARCHE
**1356**	Salesi-Ancona	F	SCN8A	1	3	MARCHE
**1357**	Salesi-Ancona	M	SCN1A	2	3	ABRUZZI
**1358**	Salesi-Ancona	F	SCN1A	0	0	MARCHE
**1359**	Salesi-Ancona	F	BRAT1	0	0	MARCHE
**1360**	Salesi-Ancona	F	PURA	0	0	ABRUZZI
**1361**	Salesi-Ancona	F	KCNQ2	0	0	MARCHE
**1362**	Salesi-Ancona	M	KCNQ2	0	0	ABRUZZI
**1363**	Salesi-Ancona	M	SCN1A	0	0	MARCHE
**1364**	Salesi-Ancona	F	MECP2	2	2	ABRUZZI
**1365**	Salesi-Ancona	F	SCN1A	2	3	ABRUZZI
**1366**	Salesi-Ancona	M	PACS2	1	2	ABRUZZI
**1367**	Salesi-Ancona	M	SCN1A	1	2	MARCHE
**1368**	Salesi-Ancona	F	PCDH19	5	13	ABRUZZI
**1369**	Salesi-Ancona	F	SLC12A5	12	14	MARCHE
**1370**	Salesi-Ancona	M	CACNA1A	3	3	.
**1371**	Salesi-Ancona	M	SCN1A	3	4	MARCHE
**1372**	Salesi-Ancona	F	SCN1A	2	10	MARCHE
**1373**	Salesi-Ancona	F	PCDH19	4	8	MARCHE
**1374**	Salesi-Ancona	M	SCN1A	29	35	ABRUZZI
**1375**	Salesi-Ancona	M	KCNQ2	2	9	MARCHE
**1376**	Salesi-Ancona	F	SCN1A	2	9	ABRUZZI
**1377**	Salesi-Ancona	M	SCN1A	6	7	ABRUZZI
**1378**	Salesi-Ancona	F	SCN1A	24	29	ABRUZZI
**1379**	Salesi-Ancona	M	CAD	28	28	UMBRIA
**1380**	Salesi-Ancona	M	WWOX	24	25	CALABRIA
**1381**	SollievoeSoff.-FG	M	GRIN2A	14	18	PUGLIE
**1382**	SollievoeSoff.-FG	M	GRIN2A	20	24	PUGLIE
**1383**	SollievoeSoff.-FG	M	SCN1A	3	7	PUGLIE
**1384**	SollievoeSoff.-FG	M	SCN1A	24	28	PUGLIE
**1385**	SollievoeSoff.-FG	M	SCN1A	17	21	PUGLIE
**1386**	SollievoeSoff.-FG	F	SCN1A	1	5	PUGLIE
**1387**	SollievoeSoff.-FG	M	KCNQ2	0	4	PUGLIE
**1388**	SollievoeSoff.-FG	F	SPTAN1	23	26	PUGLIE
**1389**	SollievoeSoff.-FG	M	STXBP1	6	9	PUGLIE
**1390**	SollievoeSoff.-FG	M	ARX	1	4	PUGLIE
**1391**	SollievoeSoff.-FG	F	MECP2	2	5	PUGLIE
**1392**	SollievoeSoff.-FG	F	KCNQ2	1	4	PUGLIE
**1393**	SollievoeSoff.-FG	F	KCNQ2	1	4	BASILICATA
**1394**	SollievoeSoff.-FG	F	CDKL5	27	30	PUGLIE
**1395**	SollievoeSoff.-FG	F	SCN1A	10	13	PUGLIE
**1396**	SollievoeSoff.-FG	F	SCN1A	3	6	PUGLIE
**1397**	SollievoeSoff.-FG	F	PCDH19	7	10	CAMPANIA
**1398**	SollievoeSoff.-FG	M	KCNQ2	21	24	BASILICATA
**1399**	SollievoeSoff.-FG	F	MECP2	4	7	CAMPANIA
**1400**	SollievoeSoff.-FG	F	ATP1A2	2	4	PUGLIE
**1401**	SollievoeSoff.-FG	F	GRIN2A	10	12	SICILIA
**1402**	SollievoeSoff.-FG	F	SCN1A	12	14	BASILICATA
**1403**	SollievoeSoff.-FG	M	SCN2A	18	20	PUGLIE

1404	SollievoeSoff.-FG	F	SCN2A	1	3	BASILICATA
1405	SollievoeSoff.-FG	M	GABRA1	12	14	PUGLIE
1406	SollievoeSoff.-FG	M	CDKL5	9	11	EMILIA ROMAGNA
1407	SollievoeSoff.-FG	F	FOXG1	54	56	SICILIA
1408	SollievoeSoff.-FG	M	SCN1A	2	4	PUGLIE
1409	SollievoeSoff.-FG	F	ATP1A2	8	10	SICILIA
1410	SollievoeSoff.-FG	M	SCN1A	5	7	PUGLIE
1411	SollievoeSoff.-FG	F	GABRA1	5	7	EMILIA ROMAGNA
1412	SollievoeSoff.-FG	F	GABRA1	20	22	PUGLIE
1413	SollievoeSoff.-FG	F	CHD2	22	24	PUGLIE
1414	SollievoeSoff.-FG	M	SLC2A1	10	12	PUGLIE
1415	SollievoeSoff.-FG	F	MECP2	26	27	PUGLIE
1416	SollievoeSoff.-FG	M	SLC2A1	13	14	SICILIA
1417	SollievoeSoff.-FG	M	SCN1A	31	32	PUGLIE
1418	SollievoeSoff.-FG	F	PCDH19	1	2	BASILICATA
1419	SollievoeSoff.-FG	M	DNM1	18	19	PUGLIE
1420	SollievoeSoff.-FG	M	SCN2A	7	8	SICILIA
1421	SollievoeSoff.-FG	M	SCN8A	5	6	PUGLIE
1422	SollievoeSoff.-FG	F	BRAT1	0	1	PUGLIE
1423	SollievoeSoff.-FG	M	CACNA1A	5	5	SICILIA
1424	SollievoeSoff.-FG	F	MECP2	6	6	SARDEGNA
1425	SollievoeSoff.-FG	M	SCN1A	4	4	PUGLIE
1426	SollievoeSoff.-FG	M	SCN1A	8	8	SICILIA
1427	SollievoeSoff.-FG	F	SCN2A	5	5	SICILIA
1428	SollievoeSoff.-FG	F	NEXMIF	37	37	PUGLIE
1429	SollievoeSoff.-FG	F	GRIN2A	11	11	PUGLIE
1430	SollievoeSoff.-FG	M	GRIN2A	11	11	SICILIA
1431	SollievoeSoff.-FG	F	SCN2A	1	1	PUGLIE
1432	SollievoeSoff.-FG	F	SCN1A	3	3	LOMBARDIA
1433	SollievoeSoff.-FG	M	SCN1A	5	5	PUGLIE
1434	SollievoeSoff.-FG	F	KCNQ2	1	4	PUGLIE
1435	SollievoeSoff.-FG	F	SCN1A	2	4	PUGLIE
1436	SollievoeSoff.-FG	F	SCN1A	44	45	PUGLIE
1437	SollievoeSoff.-FG	F	MECP2	44	45	PUGLIE
1438	SollievoeSoff.-FG	M	SCN1A	38	40	PUGLIE
1439	SollievoeSoff.-FG	M	SCN1A	31	32	PUGLIE
1440	UOCNeuropsichiatriaInfantileVerona	M	ATP1A2	9	16	VENETO
1441	UOCNeuropsichiatriaInfantileVerona	F	CDKL5	10	14	TRENTINO A. A.
1442	UOCNeuropsichiatriaInfantileVerona	M	DNM1	5	8	LOMBARDIA
1443	UOCNeuropsichiatriaInfantileVerona	F	GABRB3	1	3	TRENTINO A. A.
1444	UOCNeuropsichiatriaInfantileVerona	F	HCN1	1	5	VENETO
1445	UOCNeuropsichiatriaInfantileVerona	M	KCNQ2	0	7	VENETO
1446	UOCNeuropsichiatriaInfantileVerona	F	KCNQ2	0	8	VENETO
1447	UOCNeuropsichiatriaInfantileVerona	F	KCNQ2	2	10	TRENTINO A. A.
1448	UOCNeuropsichiatriaInfantileVerona	F	NEXMIF	2	4	VENETO
1449	UOCNeuropsichiatriaInfantileVerona	F	PCDH19	22	32	LOMBARDIA
1450	UOCNeuropsichiatriaInfantileVerona	F	PCDH19	16	26	VENETO
1451	UOCNeuropsichiatriaInfantileVerona	F	PCDH19	2	12	VENETO
1452	UOCNeuropsichiatriaInfantileVerona	F	PCDH19	8	16	CAMPANIA
1453	UOCNeuropsichiatriaInfantileVerona	F	SCN1A	3	13	LOMBARDIA
1454	UOCNeuropsichiatriaInfantileVerona	M	SCN1A	2	11	VENETO
1455	UOCNeuropsichiatriaInfantileVerona	M	SCN2A	21	23	LOMBARDIA

1456	UOCNeuropsichiatriaInfantileVerona	F	SCN2A	7	15	VENETO
1457	UOCNeuropsichiatriaInfantileVerona	M	SCN2A	12	20	EMILIA ROMAGNA
1458	UOCNeuropsichiatriaInfantileVerona	F	SCN2A	1	8	VENETO
1459	UOCNeuropsichiatriaInfantileVerona	F	SCN2A	1	8	TRENTINO A. A.
1460	UOCNeuropsichiatriaInfantileVerona	F	SCN8A	1	8	EMILIA ROMAGNA
1461	UOCNeuropsichiatriaInfantileVerona	F	SCN8A	15	21	PIEMONTE
1462	UOCNeuropsichiatriaInfantileVerona	F	SLC2A1	18	18	VENETO
1463	UOCNeuropsichiatriaInfantileVerona	M	STXBP1	3	13	LOMBARDIA
1464	UOCNeuropsichiatriaInfantileVerona	M	SYNGAP1	10	11	PIEMONTE
1465	UOCNeuropsichiatriaInfantileVerona	F	YWHAG	4	6	TRENTINO A. A.
1466	UOCNeuropsichiatriaInfantileVerona	F	ALG13	7	11	VENETO
1467	UOCNeuropsichiatriaInfantileVerona	F	ARX	9	14	CALABRIA
1468	UOCNeuropsichiatriaInfantileVerona	M	CACNA1A	9	18	VENETO
1469	UOCNeuropsichiatriaInfantileVerona	F	CACNA1A	6	12	VENETO
1470	UOCNeuropsichiatriaInfantileVerona	M	CACNA1A	11	17	TRENTINO A. A.
1471	UOCNeuropsichiatriaInfantileVerona	M	KCNT1	16	22	VENETO
1472	UOCNeuropsichiatriaInfantileVerona	F	CDKL5	16	23	PUGLIE
1473	UOCNeuropsichiatriaInfantileVerona	M	CDKL5	1	10	TOSCANA
1474	UOCNeuropsichiatriaInfantileVerona	F	CDKL5	7	14	VENETO
1475	UOCNeuropsichiatriaInfantileVerona	M	SCN1A	15	21	VENETO
1476	UOCNeuropsichiatriaInfantileVerona	F	CSNK2B	11	12	VENETO
1477	UOCNeuropsichiatriaInfantileVerona	M	GRIN2A	16	20	VENETO
1478	UOCNeuropsichiatriaInfantileVerona	M	GRIN2A	9	16	VENETO
1479	UOCNeuropsichiatriaInfantileVerona	M	GRIN2B	5	12	VENETO
1480	UOCNeuropsichiatriaInfantileVerona	M	KCNQ2	2	5	VENETO
1481	UOCNeuropsichiatriaInfantileVerona	F	SLC6A1	19	20	LOMBARDIA
1482	UOCNeuropsichiatriaInfantileVerona	M	KCNT1	10	16	TOSCANA
1483	UOCNeuropsichiatriaInfantileVerona	M	KCNT1	14	19	PIEMONTE
1484	UOCNeuropsichiatriaInfantileVerona	M	MBD5	19	25	LOMBARDIA
1485	UOCNeuropsichiatriaInfantileVerona	F	PCDH19	2	8	PUGLIE
1486	UOCNeuropsichiatriaInfantileVerona	F	SLC6A1	16	19	VENETO
1487	UOCNeuropsichiatriaInfantileVerona	F	KCNQ2	19	19	VENETO
1488	UOCNeuropsichiatriaInfantileVerona	F	SCN1A	3	7	VENETO
1489	UOCNeuropsichiatriaInfantileVerona	M	SCN1A	7	14	VENETO
1490	UOCNeuropsichiatriaInfantileVerona	M	SCN1A	47	50	VENETO
1491	UOCNeuropsichiatriaInfantileVerona	M	SCN1A	11	16	LOMBARDIA
1492	UOCNeuropsichiatriaInfantileVerona	M	SCN1A	8	15	TRENTINO A. A.
1493	UOCNeuropsichiatriaInfantileVerona	M	SCN1A	3	9	VENETO
1494	UOCNeuropsichiatriaInfantileVerona	F	SCN1A	17	24	VENETO
1495	UOCNeuropsichiatriaInfantileVerona	M	SCN1A	1	7	VENETO
1496	UOCNeuropsichiatriaInfantileVerona	F	SCN1A	3	10	LOMBARDIA
1497	UOCNeuropsichiatriaInfantileVerona	M	SCN1A	20	27	VENETO
1498	UOCNeuropsichiatriaInfantileVerona	M	SCN1A	7	11	TRENTINO A. A.
1499	UOCNeuropsichiatriaInfantileVerona	M	SCN1A	4	11	VENETO
1500	UOCNeuropsichiatriaInfantileVerona	M	SCN1A	3	8	TRENTINO A. A.
1501	UOCNeuropsichiatriaInfantileVerona	M	SCN1A	2	9	VENETO
1502	UOCNeuropsichiatriaInfantileVerona	M	SCN8A	13	19	VENETO
1503	UOCNeuropsichiatriaInfantileVerona	M	SCN1A	16	16	VENETO
1504	UOCNeuropsichiatriaInfantileVerona	F	SCN1A	28	35	UMBRIA
1505	UOCNeuropsichiatriaInfantileVerona	F	SCN1A	31	36	VENETO
1506	UOCNeuropsichiatriaInfantileVerona	M	SCN1A	8	16	LAZIO
1507	UOCNeuropsichiatriaInfantileVerona	F	SCN1A	16	19	VENETO

1508	UOCNeuropsichiatriaInfantileVerona	M	SCN1A	4	12	VENETO
1509	UOCNeuropsichiatriaInfantileVerona	F	SCN1A	42	48	PIEMONTE
1510	UOCNeuropsichiatriaInfantileVerona	F	SCN1A	7	14	LOMBARDIA
1511	UOCNeuropsichiatriaInfantileVerona	M	SPTAN1	17	18	VENETO
1512	UOCNeuropsichiatriaInfantileVerona	M	SCN1A	21	28	VENETO
1513	UOCNeuropsichiatriaInfantileVerona	F	SCN1A	5	11	VENETO
1514	UOCNeuropsichiatriaInfantileVerona	F	SCN1A	14	16	VENETO
1515	UOCNeuropsichiatriaInfantileVerona	F	SCN2A	14	21	SICILIA
1516	UOCNeuropsichiatriaInfantileVerona	M	SCN2A	12	19	SICILIA
1517	UOCNeuropsichiatriaInfantileVerona	M	SCN2A	33	33	VENETO
1518	UOCNeuropsichiatriaInfantileVerona	M	SCN1A	15	17	VENETO
1519	UOCNeuropsichiatriaInfantileVerona	M	SCN2A	74	76	VENETO
1520	UOCNeuropsichiatriaInfantileVerona	M	SCN8A	3	6	VENETO
1521	UOCNeuropsichiatriaInfantileVerona	M	SLC2A1	3	11	VENETO
1522	UOCNeuropsichiatriaInfantileVerona	M	SLC2A1	40	48	VENETO
1523	UOCNeuropsichiatriaInfantileVerona	M	SLC2A1	5	13	VENETO
1524	UOCNeuropsichiatriaInfantileVerona	M	SLC2A1	4	12	VENETO
1525	UOCNeuropsichiatriaInfantileVerona	F	SLC6A1	10	12	VENETO
1526	UOCNeuropsichiatriaInfantileVerona	F	SPTAN1	12	18	EMILIA ROMAGNA
1527	UOCNeuropsichiatriaInfantileVerona	F	SYNGAP1	15	15	VENETO
1528	UOCNeuropsichiatriaInfantileVerona	M	CDKL5	7	16	EMILIA ROMAGNA
1529	UOCNeuropsichiatriaInfantileVerona	M	GRIN2A	13	16	PUGLIE
1530	UOCNeuropsichiatriaInfantileVerona	F	SMC1A	13	16	VENETO
1531	UOCNeuropsichiatriaInfantileVerona	M	UBA5	12	15	VENETO
1532	UOCNeuropsichiatriaInfantileVerona	F	SLC2A1	8	15	VENETO
1533	UOCNeuropsichiatriaInfantileVerona	F	MBD5	8	14	VENETO
1534	UOCNeuropsichiatriaInfantileVerona	M	PNPO	8	14	VENETO
1535	UOCNeuropsichiatriaInfantileVerona	M	SYNGAP1	11	14	VENETO
1536	UOCNeuropsichiatriaInfantileVerona	F	SLC6A1	10	13	EMILIA ROMAGNA
1537	UOCNeuropsichiatriaInfantileVerona	M	SLC6A1	10	13	VENETO
1538	UOCNeuropsichiatriaInfantileVerona	F	GABRA1	8	12	VENETO
1539	UOCNeuropsichiatriaInfantileVerona	M	SCN1A	6	12	CALABRIA
1540	UOCNeuropsichiatriaInfantileVerona	M	KCNQ2	6	12	VENETO
1541	UOCNeuropsichiatriaInfantileVerona	F	CHD2	10	12	VENETO
1542	UOCNeuropsichiatriaInfantileVerona	F	CDKL5	6	11	LAZIO
1543	UOCNeuropsichiatriaInfantileVerona	M	PURA	7	10	VENETO
1544	UOCNeuropsichiatriaInfantileVerona	M	SCN1A	7	10	EMILIA ROMAGNA
1545	UOCNeuropsichiatriaInfantileVerona	F	SCN1A	6	9	LOMBARDIA
1546	UOCNeuropsichiatriaInfantileVerona	F	KCNQ2	6	9	VENETO
1547	UOCNeuropsichiatriaInfantileVerona	F	KCNB1	5	9	VENETO
1548	UOCNeuropsichiatriaInfantileVerona	F	SCN2A	5	9	TRENTINO A. A.
1549	UOCNeuropsichiatriaInfantileVerona	F	CHD2	6	8	VENETO
1550	UOCNeuropsichiatriaInfantileVerona	M	SCN2A	2	8	.
1551	UOCNeuropsichiatriaInfantileVerona	M	CHD2	8	8	TRENTINO A. A.
1552	UOCNeuropsichiatriaInfantileVerona	M	SCN2A	1	7	LOMBARDIA
1553	UOCNeuropsichiatriaInfantileVerona	F	CDKL5	1	7	VENETO
1554	UOCNeuropsichiatriaInfantileVerona	F	NEXMIF	5	6	CAMPANIA
1555	UOCNeuropsichiatriaInfantileVerona	M	STXBP1	2	5	VENETO
1556	UOCNeuropsichiatriaInfantileVerona	F	STXBP1	1	5	VENETO
1557	UOCNeuropsichiatriaInfantileVerona	F	SCN2A	1	4	VENETO
1558	UOCNeuropsichiatriaInfantileVerona	M	HNRNPU	2	4	VENETO
1559	UOCNeuropsichiatriaInfantileVerona	M	KCNQ2	0	3	VENETO

**1560**	UOCNeuropsichiatriaInfantileVerona	M	SLC9A6	3	3	VENETO
**1561**	UOCNeuropsichiatriaInfantileVerona	F	PCDH19	2	3	VENETO
**1562**	UOCNeuropsichiatriaInfantileVerona	M	RHOBTB2	0	0	VENETO
**1563**	UOCNeuropsichiatriaInfantileVerona	F	SCN2A	0	0	VENETO
**1564**	UOCNeuropsichiatriaInfantileVerona	F	CDKL5	0	0	VENETO
**1565**	UOCNeuropsichiatriaInfantileVerona	F	CDKL5	0	0	VENETO
**1566**	UOCNeuropsichiatriaInfantileVerona	M	SCN1A	39	42	SICILIA
**1567**	UOCNeuropsichiatriaInfantileVerona	F	CDKL5	28	35	TRENTINO A. A.
**1568**	UOCNeuropsichiatriaInfantileVerona	M	STXBP1	19	23	VENETO
